# Discovery of dual kinase inhibitors targeting VEGFR2 and FAK: structure-based pharmacophore modeling, virtual screening, and molecular docking studies

**DOI:** 10.1186/s13065-024-01130-5

**Published:** 2024-02-12

**Authors:** Marwa A. Fouad, Alaa A. Osman, Noha M. Abdelhamid, Mai W. Rashad, Ashrakat Y. Nabawy, Ahmed M. El Kerdawy

**Affiliations:** 1 Pharmaceutical Chemistry Department, Faculty of Pharmacy, Cairo University, Kasr El-Aini St., Cairo, 11562 Egypt; 2 Pharmaceutical Chemistry Department, School of Pharmacy, Newgiza University (NGU), Newgiza, Km 22 Cairo-Alexandria Desert Road, Cairo, Egypt; 3 School of Pharmacy, College of Health and Science, University of Lincoln, Joseph Banks Laboratories, Green Lane, Lincoln, Lincolnshire UK

**Keywords:** Pharmacophore modelling, Virtual screening, Molecular docking, Cancer, FAK, VEGFR2, Angiogenesis, Multi-kinase inhibitors

## Abstract

VEGFR2 and FAK signaling pathways are interconnected and have synergistic effects on tumor angiogenesis, growth, and metastasis. Thus, instead of the conventional targeting of each of these proteins individually with a specific inhibitor, the present work aimed to discover novel dual inhibitors targeting both VEGFR2 and FAK exploiting their association. To this end, receptor-based pharmacophore modeling technique was opted to generate 3D pharmacophore models for VEGFR2 and FAK type II kinase inhibitors. The generated pharmacophore models were validated by assessing their ability to discriminate between active and decoy compounds in a pre-compiled test set of VEGFR2 and FAK active compounds and decoys. ZINCPharmer web tool was then used to screen the ZINC database purchasable subset using the validated pharmacophore models retrieving 42,616 hits for VEGFR2 and 28,475 hits for FAK. Subsequently, they were filtered using various filters leaving 13,023 and 6,832 survived compounds for VEGFR2 and FAK, respectively, with 124 common compounds. Based on molecular docking simulations, thirteen compounds were found to satisfy all necessary interactions with VEGFR2 and FAK kinase domains. Thus, they are predicted to have a possible dual VEGFR2/FAK inhibitory activity. Finally, SwissADME web tool showed that compound ZINC09875266 is not only promising in terms of binding pattern to our target kinases, but also in terms of pharmacokinetic properties.

**Supplementary Information:**

The online version contains supplementary material available at 10.1186/s13065-024-01130-5.

## Introduction

Research on anticancer agents began in the twentieth century, yet the development of efficient, safe, and selective anticancer agents remains a research hotspot [1]. Conventional chemotherapeutic agents cause unfavorable side effects due to their lack of selectivity towards cancer cells over normal cells. On the other hand, targeted therapies, such as anti-angiogenic agents, show a higher selectivity towards cancer cells or their supporting microenvironment, thus, with minimum side effects on normal cells [2].

Angiogenesis, which involves the formation of new blood vessels from pre-existing ones, is a key biological process involved in many physiological as well as pathological conditions [3, 4]. Physiologically, angiogenesis is important for the development of embryos, menstruation, and wound healing, however, it is also an integral part of several diseases such as cancer [5, 6]. The process of solid tumor growth inevitably involves angiogenesis as a means for delivering oxygen and nutrients to the continuously growing tumor cells [7]. This process is vital for the growing of primary tumors beyond the size of 1–2 mm^3^, as well as for their spread and metastasis [8]. Thus, angiogenesis induction represents one of the key cancer hallmarks that are shared by all types of cancer [9, 10]. Angiogenesis is tightly regulated through the balance between stimulatory (proangiogenic) and inhibitory (antiangiogenic) signals, a phenomenon known as the angiogenic switch [11]. This switch is considered “on” when the proangiogenic signals overpower those of the antiangiogenic signals [12].

Cancer treatment strategies based on targeting tumor angiogenesis demonstrated a great potential in curbing tumor growth and metastasis which is one of the biggest contributing factors to mortality in cancer patients [13, 14]. The benefit of anti-angiogenic agents in cancer patients’ survival is still under investigation; however, it has been demonstrated in several clinical trials that the combination of anti-angiogenic agents with a cytotoxic chemotherapy led to an increase in patients’ overall survival (OS) and progression free survival (PFS) [15].

Protein kinases (PKs) are critical mediators and coordinators of several cellular signaling pathways involved in cell proliferation, differentiation, migration, survival, and apoptosis [16]. There are two major classes of PKs; protein tyrosine kinases (PTK), and serine/threonine kinases (STK), phosphorylating tyrosine residues and serine/threonine residues, respectively, in the substrate proteins [17–20]. PTKs are further divided into two subfamilies, receptor tyrosine kinases (RTK) such as vascular endothelial growth factor receptor (VEGFR) and non-receptor tyrosine kinases (NRTK) such as focal adhesion kinase (FAK) [21, 22].

PTKs play an important role in the process of angiogenesis. In the context of oncology, several PTKs directly regulate tumor angiogenesis including fibroblast growth factor receptor (FGFR), platelet-derived growth factor receptor (PDGFR), and vascular endothelial growth factor receptor (VEGFR) which has three isoforms; VEGFR-1 (Flt-1), VEGFR2 (KDR), and VEGFR-3 (Flt-4) [23]. In vascular endothelial cells, VEGFR2 is the major signaling VEGFR and it plays a significant role in the process of tumor angiogenesis [24–26]. Moreover, VEGFR2 overexpression has been reported in a variety of cancers such as esophageal, oral, ovarian, and prostate cancer [27, 28].

Focal adhesion kinase (FAK) is a NRTK that has a key role in angiogenesis as it regulates endothelial cells and fibroblasts migration and invasion which is an integral part of angiogenesis [29]. FAK overexpression can be traced to many pathological conditions, especially cancer [30]. It is overexpressed in several cancer types such as head and neck [31], oral [32], thyroid [33], cervical [34], ovarian [35], breast [36], colon [36], and prostate cancer [37]. Besides its direct effect on angiogenesis, FAK increased expression in cancer cells plays a key role in the tumor angiogenic switch promoting aggressive tumor progression and metastasis [38]. Moreover, FAK inhibitors were found to suppress tumor growth and tumor vascular formation in animal models [39].

Several studies reported the association between VEGFR2 and FAK [29, 39–43]. FAK is stimulated by several angiogenic growth factor receptors including VEGFR2 when stimulated by VEGF-A [29, 40]. Moreover, FAK forms an integrin αvβ5 signaling complex in a Src-dependent manner which is essential for VEGF stimulated angiogenesis [41, 42]. Furthermore, VEGFR2 and FAK were found to be positively correlated in patients with triple negative breast cancer (TNBC), in addition, FAK promotes angiogenesis in TNBC cells through regulating VEGFR2 and VEGF protein expression [39]. Furthermore, inhibition of FAK expression in neuroblastoma, breast, and prostate carcinoma cells results in reduced VEGF expression [43].

Protein kinase inhibition can be achieved by different types of inhibitors, such as small molecule inhibitors and monoclonal antibodies [2]. There are several types of small molecule PK inhibitors (I–VI) based on the nature of the inhibitor and its binding interactions, the site of ligand binding, and the conformation of the PK-ligand complex formed [44]. Type II ATP-competitive inhibitors bind to the DFG-out inactive kinase conformation occupying the hinge region (Front pocket) and extend through the gate area towards the hydrophobic allosteric back pocket [45, 46]. They have proven to be better drug candidates and more advantageous over those of type I as they have higher affinity and selectivity [46, 47]. Moreover, they possess slower dissociation rates (≈10 times slower) and so longer residence time leading to longer suppression of kinase activity [48, 49].

**Fig. 1:**
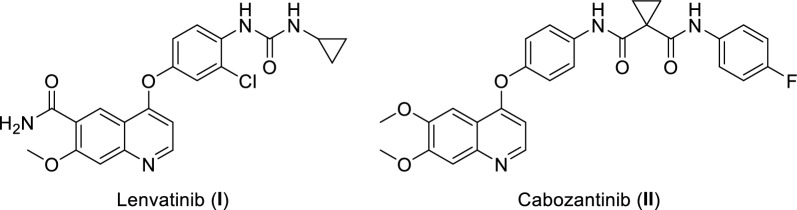
Examples for FDA approved multi-kinase inhibitors

Multi-kinase inhibitors supersede single kinase inhibitors in many aspects. For starters, as they inhibit two or more proteins, they have a resultant synergistic effect, which in turn results in a greater potency [50]. They also show enhanced pharmacokinetic characteristics and a reduced risk of developing resistance [51]. There are already a few multi-kinase inhibitors that have received FDA approval such Lenvatinib (**I**) which inhibits VEGFR1, VEGFR2, and VEGFR3, and Cabozantinib (**II**) which inhibits c-Met and VEGFR2 (Fig. 1) [52].

**Fig. 2:**
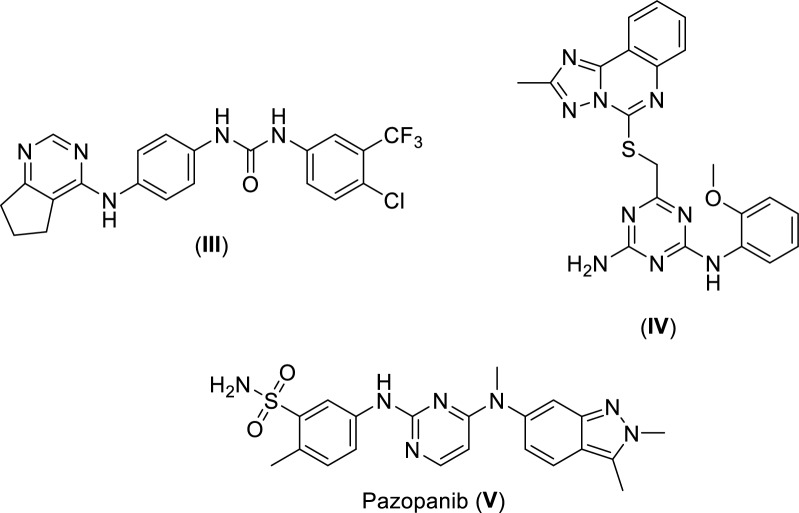
Examples for kinase inhibitors discovered using CADD approaches

Throughout the past years, considerable progress has been made in the discovery of protein kinase inhibitors, and this goes back to the use of computational methods [53, 54]. The two main approaches of computer-aided drug design (CADD), ligand-based drug design (LBDD) and structure-based drug design (SBDD), provide valuable tools for studying the different protein kinase structures and designing kinase inhibitors [53–55]. For example, LBDD represented by 3D QSAR pharmacophore model for VEGFR2 inhibitors was used to virtually screen different databases for novel hits resulting in the discovery of the 6,7-dihydro-5H-cyclopenta[d]pyrimidine derivative (**III**) (Fig. 2) as a promising VEGFR2 inhibitor with IC_50_ of 0.85 µM [56]. Moreover, ligand-based pharmacophore model for FAK inhibitors was used to virtually screen ZINC database identifying compound (**IV**) (Fig. 2) as a potential hit [57]. On the other hand, the discovery of the clinically approved VEGFR2 inhibitor, pazopanib (**V**) (Fig. 2), is an example of the use of homology modeling and SBDD for the design of kinase inhibitors [58, 59].

As pointed out earlier, VEGFR2 and FAK signaling pathways are interconnected and have synergistic effects on angiogenesis, tumor growth, and metastasis [29, 39–43]. Thus, instead of targeting each of these proteins individually with a specific inhibitor, the present work aims to discover novel type II dual inhibitors simultaneously targeting VEGFR2 and FAK exploiting their association. This approach could provide a more comprehensive targeting of angiogenesis, tumor progression and metastasis which could lead to improved treatment outcomes. In addition, it could be a possible strategy to overcome resistance mechanisms that arise from single-target inhibition. The simultaneous targeting of multiple pathways can make it more difficult for cancer cells to develop resistance mechanisms, potentially prolonging the effectiveness of the therapy.

To this end, a training set of VEGFR2 and FAK protein structures bound to type II inhibitors retrieved from the Protein Data Bank (PDB) (https://www.rcsb.org) will be used. Receptor-based pharmacophore models will then be manually generated based on the common interactions extracted from the co-crystalized inhibitors in each protein. The generated pharmacophore models for each kinase will be filtered and validated utilizing compiled test sets of VEGFR2/FAK inhibitors and decoys. The pharmacophore model survives the filtration and validation step for each protein will be then used to screen the ZINC purchasable database. Hits recovered from the virtual screening will be filtered to keep only promising lead-like compounds with acceptable pharmacokinetic properties. Common molecules survive the filtration step in both proteins will be then subjected to molecular docking simulations. Molecules’ docking poses will be then evaluated to extract molecules that bind in both proteins’ kinase domains performing the essential interactions.

## Results and discussion

### Protein structure similarity assessment

To assess the rationale of our novel strategy and the potential of finding dual inhibitors, the similarity of VEGFR2 and FAK kinase domains in sequence, topology, and structure was initially investigated. VEGFR2 and FAK kinase domain amino acid sequences were obtained in FASTA format from the Protein Data Bank (VEGFR2-PDB-ID: 4ASD [60] and FAK-PDB-ID: 4K9Y [61]) (https://www.rcsb.org). Then, NCBI Basic Local Alignment Search Tool for proteins (BLASTp) (https://blast.ncbi.nlm.nih.gov/Blast.cgi) was used to align and assess the similarity between the amino acid sequences using VEGFR2 kinase domain sequence as the query and FAK kinase domain sequence as the subject.

**Fig. 3:**
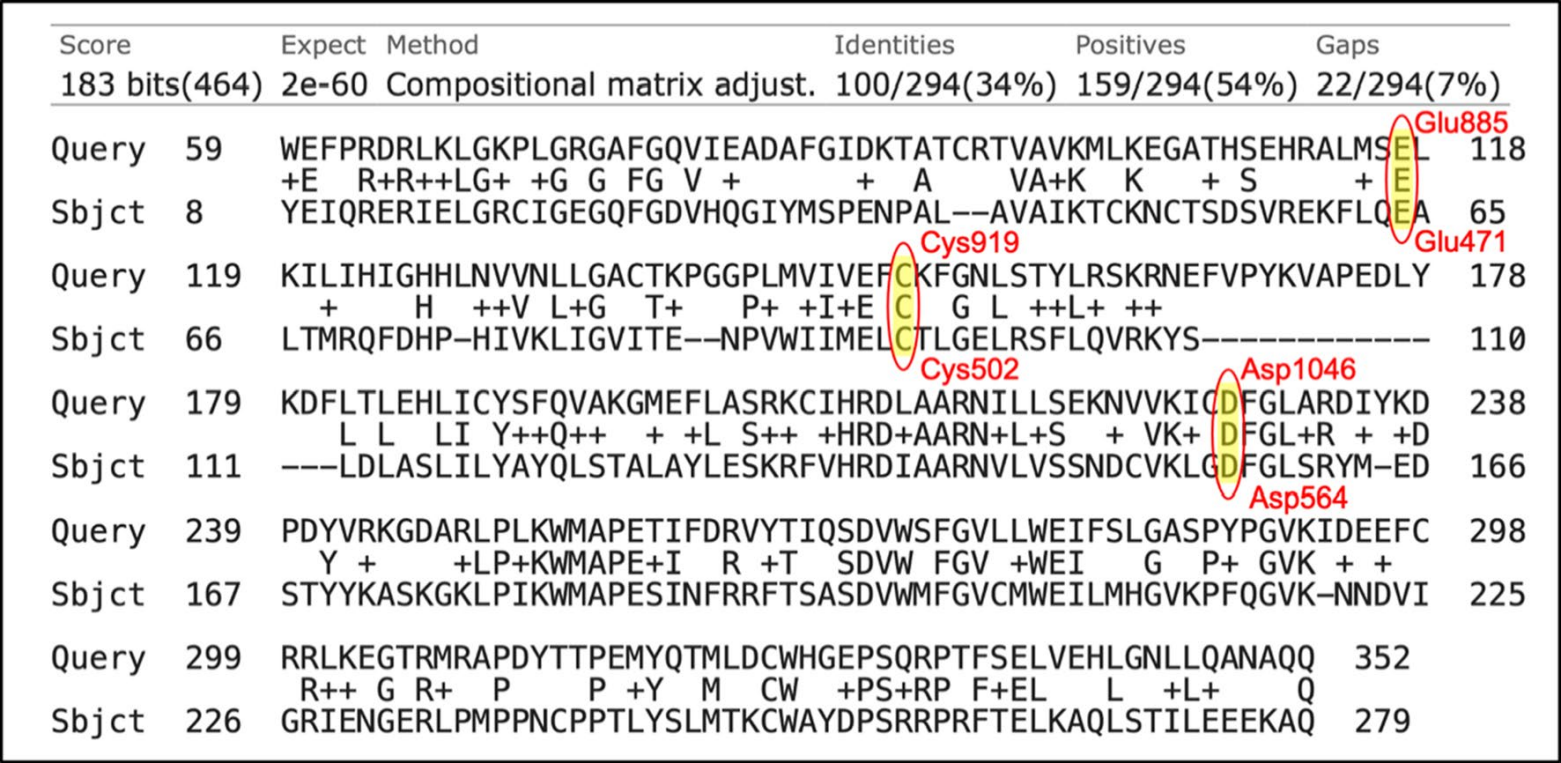
VEGFR2 (Query) and FAK (Subject) kinase domains amino acid sequence alignment using BLASTp (Aligned residues involved in type II kinase inhibition binding pattern are highlighted)

**Fig. 4:**
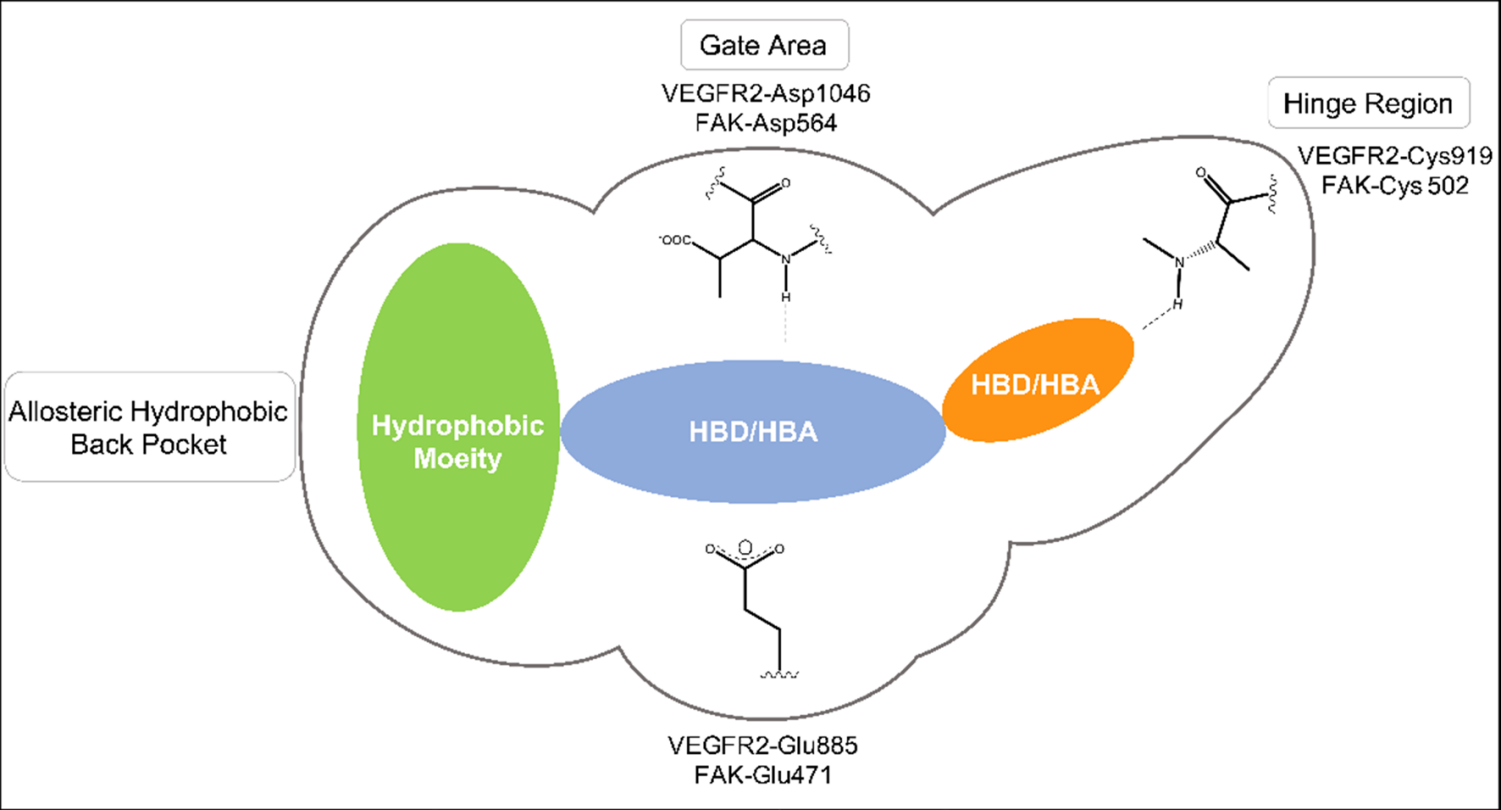
Key structural features of type II kinase inhibitors

Upon sequence alignment, a promising sequence similarity of 34% was found (Fig. 3), moreover, the percentage of positives was 54%, indicating a great portion of sequence differences was due to conservative substitutions, with the replaced amino acids having side chains with similar nature. Furthermore, the amino acid residues involved in type II kinase inhibition binding pattern were found to align, these include hinge region cysteine, αC helix glutamic acid, and the DFG loop aspartic acid (Figs. 3 and 4).

**Fig. 5.**
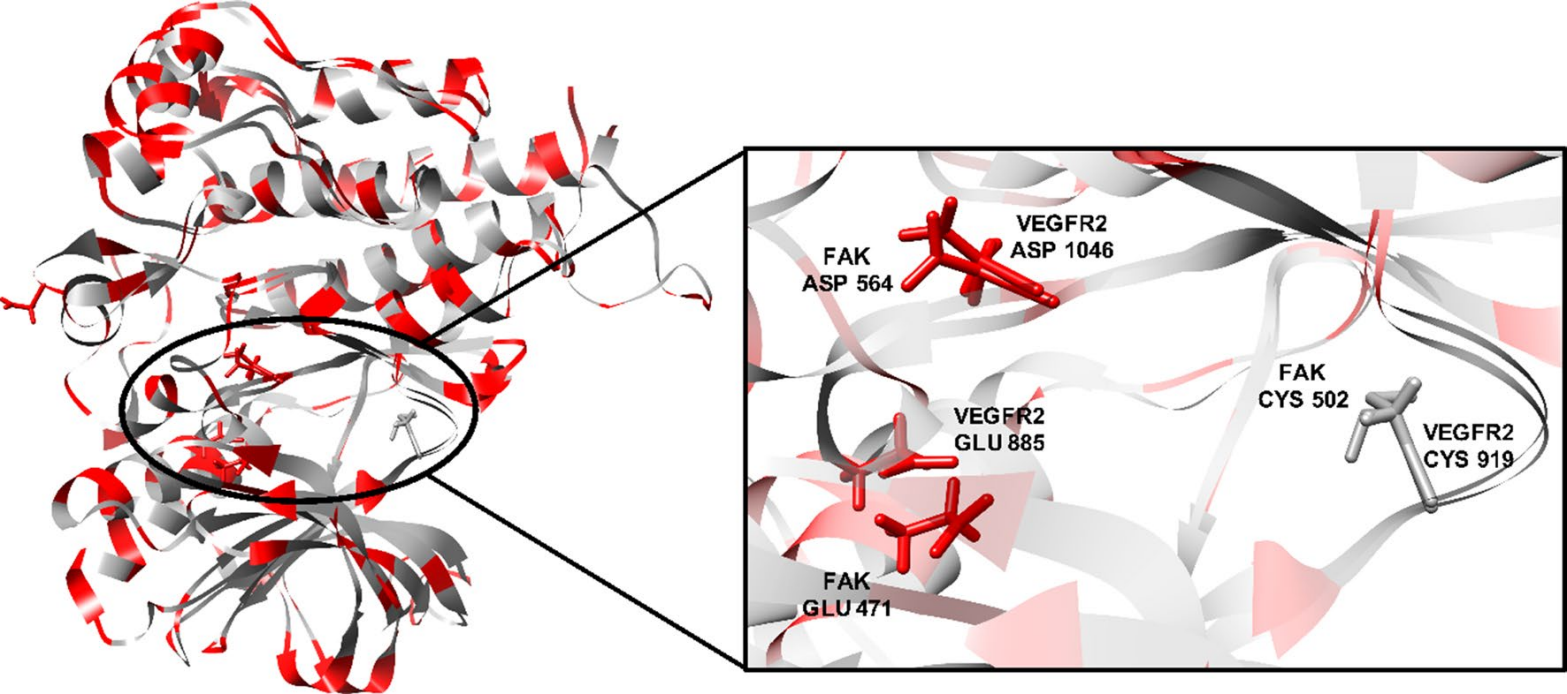
3D superimposition of VEGFR2 (PDB ID: 4ASD) and FAK (PDB ID: 4K9Y) protein structures with a close focus on the key residues at the ATP binding site (Hydrophobic region: grey; Polar regions: red)

Furthermore, VEGFR2 and FAK crystal structures (PDB ID: 4ASD [60] and PDB ID: 4K9Y [61], respectively) were downloaded from the protein data bank (PDB) (https://www.rcsb.org/). Then, they were aligned, and their overlay was investigated, especially, the ATP-binding site and its key amino acids. As can be seen in Fig. 5, the obtained alignment shows that the protein structures are well aligned with the proteins’ hydrophobic and polar regions superimposed, in particular, the key residues at the ATP-binding site. The key binding residues are not only showing high similarity in their position within the binding pocket in 3D space but also in their type and nature. The hinge region residue, Cys919 in VEGFR2 aligns with Cys502 in FAK, the αC helix Glu885 in VEGFR2 and Glu471 in FAK are also found to converge in 3D space, and the DFG loop Asp1046 in VEGFR2 aligns with Asp564 in FAK. Thus, it is evident that the key regions for inhibitor design represented by the hinge region, the gate area, and the hydrophobic back pocket show high topological and residue similarity.

These results indicate that VEGFR2 and FAK are not only related to cancer angiogenesis, growth, and metastasis but also with similar kinase domains in sequence, topology, and structure and so the possibility of finding inhibitors that can target both kinase domains simultaneously (Dual inhibitors) is an amenable task.

### Retrieving X-ray crystallographic structures and training set generation

The active site of protein kinases could be divided into three sub-regions: the hinge region (Front pocket), the gate area and the hydrophobic allosteric back pocket. The hinge region is a flexible coil, which resides between the N-terminal and the C-terminal lobes. The hydrophobic allosteric back pocket is exposed in the DFG-out conformation [62]. To accomplish type-II-like dual inhibition, the inhibitors should retain hydrogen-bonding interactions with the hinge region, gate area, and hydrophobic interactions with the hydrophobic back pocket in both VEGFR2 and FAK kinase domains [63].

In the current work, for dual VEGFR2/FAK type II inhibitor design, inhibitors need to perform the crucial hydrogen bond interactions with the VEGFR2 Cys919 and FAK Cys502 residues at the hinge region [44, 64, 65]. Additionally, several interactions via hydrogen bonds with VEGFR2 Asp1046 and FAK Asp564 of the conserved DFG motif as well as VEGFR2 Glu885 and FAK Glu471 of αC helix at the interface between gate area and the hydrophobic back pocket, and finally they should extend beyond the gate area to interact through hydrophobic interaction with the allosteric back pocket exposed in DFG-out conformation [25, 61, 64, 65]. Commonly, additional interactions with other residues at the binding site would strengthen the binding affinity [66].

Ten X-ray crystallographic structures of VEGFR2 (8) and FAK (2) co-crystallized with different type II inhibitors (VEGFR2-PDB ID: 4ASE, 4ASD, 2QU6, 3VHE, 3EWH, 3VNT, 3WZD, and 6XVK; FAK-PDB ID: 4KAO and 4K9Y) [47, 60, 61, 67–71] were downloaded from the Protein Data Bank (https://www.rcsb.org/) (Tables 1 and 2). Structural diversity was kept in mind while constructing our training set, moreover, it was ensured that all compounds included in our training set were able to perform all the essential interactions with the font cleft, gate area, and hydrophobic back pocket (Tables 1 and 2). For further details about training set compounds, see Additional file 1; S1. Training set compounds for VEGFR2 and FAK pharmacophore model generation.

**Table 1:** VEGFR2 PDB structures used in training set generation

#	PDB ID	Ref	Ligand structure	Ligand/protein interactions	IC_50_ (nM)
VEGFR_1	4ASE	[60]			0.04
VEGFR_2	4ASD	[60]			2.3
VEGFR_3	2QU6	[67]			15
VEGFR_4	3VHE	[68]			6.2
VEGFR_5	3EWH	[69]			69 ± 10
VEGFR_6	3VNT	[70]			2.2
VEGFR_7	3WZD	[47]			5.1
VEGFR_8	6XVK	[71]			0.538

**Table 2:** FAK PDB structures used in training set generation

#	PDB ID	Ref	Ligand structure	Ligand/protein interactions	IC_50_ (nM)
FAK_1	4K9Y	[61]			266
FAK_2	4KAO	[61]			7000

### Pharmacophore model generation

3D pharmacophore models are commonly used as a virtual screening tool to obtain a more concise list of hits with a considerable complementarity to the desired targets. In the current work, receptor-based pharmacophore modeling was adopted to generate several pharmacophore models for the inhibitors of each protein kinase. The retrieved and prepared protein structures co-crystallized with various inhibitors for each protein kinase VEGFR2 and FAK (VEGFR2 PDB IDs: 4ASE, 4ASD, 2QU6, 3VHE, 3EWH, 3VNT, 3WZD, and 6XVK. FAK PDB IDs: 4KAO and 4K9Y) [47, 60, 61, 67–71] were first aligned separately. Several manual 3D pharmacophores were created for each protein using the aligned set of proteins and co-crystallized ligands featuring an extensive variety of the key pharmacophoric features (recognition, shape, and site points) for type II binding pattern. Furthermore, excluded volumes were used to mimic the actual binding constraints by defining the steric extent of the amino acid residues lining the kinase binding sites. This resulted in 109 and 14 pharmacophore models for VEGFR2 and FAK inhibitors, respectively, that are qualitatively and quantitively different, in terms of features’ type, size, and position.

### Test set compilation

To assess the performance of the generated pharmacophore models for each protein kinase inhibitors in discriminating between active inhibitors and inactive compounds, a test set of active inhibitors and decoys was constructed for each protein kinase to test and validate the different manually generated pharmacophore models. This test set contains 2240 compounds, including 1240 compounds for VEGFR2 (39 active inhibitors (see Additional file 1: Table S1. VEGFR2 test set active compounds) and 1200 decoys) and 1000 compounds for the FAK (17 active inhibitors (see additional file 1: Table S2. FAK manually collected test set compounds) and 983 decoys). The test set was constructed so that it has a large decoy/active ratio (≈30:1) in efforts to mimic the natural chemical space ratio between active and inactive compounds. Conformational search was then carried out resulting in 43,038 conformers for VEGFR2 test set compounds and 33,362 conformers for FAK test set compounds, which were then used for pharmacophore model selection and validation.

### Pharmacophore selection and validation

Based on the pharmacophore models’ capacity to discriminate between active and decoy compounds efficiently, the best models were selected from the various generated models. This was determined with the help of the compiled test set. As means of evaluating the different generated pharmacophore models, various assessment metrics (**Se**, **Sp**, **Ya**, **E**, **acc**, **DR**, and **F1**) were calculated for each one using the results of the pharmacophore models application on the test set (**TP**, **FP**, **TN**, and **FN**) (for further details see Additional file 1: Table S3. Assessment metrics of pharmacophore models performance) [72]. F1 score describes the overall model quality in discriminating between active and inactive compounds and so was used as the metric of choice for best model selection.

Regarding the generated VEGFR2 pharmacophore models, Table 3 shows that some models such as VEGFR2_Ph4_95 and VEGFR2_Ph4_105 showed a high selection potential towards true positives (TP), with sensitivity values of 0.98 and 0.98, respectively. However, they showed low accuracy of 0.09 and 0.82, respectively, as they yielded a high number of false positives (FP) which indicates their lack of specificity (0.06 and 0.81, respectively) and their bias towards active compounds. On the contrary, other models such as VEGFR2_Ph4_17, VEGFR2_Ph4_21, VEGFR2_Ph4_86, VEGFR2_Ph4_92 models showed high specificity values (1.00, 1.00, 0.98, and 1.00, respectively) but they exhibited poor sensitivity (0.08, 0.00, 0.08, and 0.05, respectively), which means that they are very efficient in identifying true negative (TN) compounds but have a very weak true positives’ sensitivity indicating their bias towards decoy compounds. Models VEGFR2_Ph4_103, VEGFR2_Ph4_104, and VEGFR2_Ph4_109 showed a balanced promising sensitivity (0.95) and specificity (0.94, 0.94, and 0.93, respectively) and so showing no bias towards actives nor decoys and so showed the highest F1 score values (0.50, 0.52, and 0.48, respectively). For the performance of all VEGFR2 pharmacophore models see Additional file 1: Table S4. VEGFR2 pharmacophore model assessment.

**Table 3:** VEGFR2 pharmacophore model assessment for representative models, in bold is the selected pharmacophore model (see Additional file 1, for all models’ results, Table S4)

Ph4-no	N	TP	FP	TN	FN	Se	Sp	Ya	E	Acc	Dr	F1
VEGFR2_Ph4-6	64	7	24	1176	33	0.18	0.98	0.11	3.39	0.95	0.18	0.2
VEGFR2_Ph4-11	223	28	183	1017	12	0.70	0.85	0.13	3.89	0.84	0.83	0.22
VEGFR2_Ph4-12	63	16	23	1177	24	0.40	0.98	0.25	7.87	0.96	0.41	0.41
VEGFR2_Ph4-17	44	3	4	1196	37	0.08	1.00	0.07	2.11	0.97	0.08	0.13
VEGFR2_Ph4-19	251	30	211	989	10	0.75	0.82	0.12	3.71	0.82	0.91	0.21
VEGFR2_Ph4-21	41	0	1	1199	40	0.00	1.00	0.00	0.00	0.97	0.00	0.00
VEGFR2_Ph4-86	66	3	26	1174	37	0.08	0.98	0.05	1.41	0.95	0.08	0.09
VEGFR2_Ph4-92	44	2	4	1196	38	0.05	1.00	0.05	1.41	0.97	0.05	0.09
VEGFR2_Ph4-95	1164	39	1124	76	1	0.98	0.06	0.03	1.04	0.09	15.39	0.06
VEGFR2_Ph4-99	140	38	100	1100	2	0.95	0.92	0.27	8.41	0.92	1.04	0.43
VEGFR2_Ph4-100	116	38	76	1124	2	0.95	0.94	0.33	10.16	0.94	1.01	0.49
VEGFR2_Ph4-101	123	39	83	1117	1	0.98	0.93	0.32	9.83	0.93	1.05	0.48
VEGFR2_Ph4-102	134	38	94	1106	2	0.95	0.92	0.28	8.79	0.92	1.03	0.44
VEGFR2_Ph4-103	113	38	73	1127	2	0.95	0.94	0.34	10.42	0.94	1.01	0.50
VEGFR2_Ph4-104	109	38	69	1131	2	0.95	0.94	0.35	10.81	0.94	1.01	0.52
VEGFR2_Ph4-105	264	39	224	976	1	0.98	0.81	0.15	4.58	0.82	1.2	0.26
VEGFR2_Ph4-106	225	38	185	1015	2	0.95	0.85	0.17	5.24	0.85	1.12	0.29
VEGFR2_Ph4-107	201	38	161	1039	2	0.95	0.87	0.19	5.86	0.87	1.1	0.32
VEGFR2_Ph4-108	162	38	122	1078	2	0.95	0.90	0.23	7.27	0.9	1.06	0.38
VEGFR2_Ph4-109	**122**	**38**	**82**	**1118**	**2**	**0.95**	**0.93**	**0.31**	**9.66**	**0.93**	**1.02**	**0.48**

As for the generated FAK pharmacophore models, it can be seen in Table 4 that FAK_Ph4_2 and FAK_Ph4_6 showed high sensitivity (1.00 and 0.94, respectively) meaning that they yielded a high number of true positives, however, they showed poor specificity (0.46 and 0.81, respectively) and so could not discard decoys and consider them as active compounds (Biased towards active compounds). On the contrary, models FAK_Ph4_4, FAK_Ph4_8, FAK_Ph4_9, and FAK_Ph4_10 showed high specificity (0.99) but low sensitivity (0.71, 0.65, 0.82 and 0.82, respectively) so, they tend to discard all compounds and consider them inactive even the true actives (Biased towards inactive compounds). Three pharmacophore models FAK_Ph4_9, FAK_Ph4_10, and FAK_Ph4_12 showed a balanced promising sensitivity (0.82, 0.82, and 0.88) and specificity (0.99, 0.99, and 0.98, respectively) and so showing no bias towards actives nor decoys and had the highest F1 scores (0.65, 0.65, and 0.61, respectively), meaning that they had the best overall performance and quality.

**Table 4:** FAK pharmacophore model assessment, in bold is the selected pharmacophore model

Ph4-no	N	TP	FP	TN	FN	Se	Sp	Ya	E	Acc	Dr	F1
FAK_Ph4-1	64	15	49	934	2	0.88	0.95	0.23	13.79	0.95	0.93	0.37
FAK_Ph4-2	549	17	532	451	0	1.00	0.46	0.03	1.82	0.47	2.18	0.06
FAK_Ph4-3	42	13	29	954	4	0.76	0.97	0.31	18.21	0.97	0.79	0.44
FAK_Ph4-4	24	12	12	971	5	0.71	0.99	0.50	29.41	0.98	0.71	0.59
FAK_Ph4-5	35	13	22	961	4	0.76	0.98	0.37	21.85	0.97	0.78	0.50
FAK_Ph4-6	204	16	188	795	1	0.94	0.81	0.08	4.61	0.81	1.16	0.14
FAK_Ph4-7	42	13	29	954	4	0.76	0.97	0.31	18.21	0.97	0.79	0.44
FAK_Ph4-8	23	11	12	971	6	0.65	0.99	0.48	28.13	0.98	0.66	0.55
FAK_Ph4-9	26	14	12	971	3	0.82	0.99	0.54	31.67	0.99	0.83	0.65
FAK_Ph4-10	26	14	12	971	3	0.82	0.99	0.54	31.67	0.99	0.83	0.65
FAK_Ph4-11	45	12	33	950	5	0.71	0.97	0.27	15.69	0.96	0.73	0.39
FAK_Ph4-12	**32**	**15**	**17**	**966**	**2**	**0.88**	**0.98**	**0.47**	**27.57**	**0.98**	**0.90**	**0.61**
FAK_Ph4-13	191	15	176	807	2	0.88	0.82	0.08	4.62	0.82	1.07	0.14
FAK_Ph4-14	55	15	40	943	2	0.88	0.96	0.27	16.04	0.96	0.92	0.42

Generally, pharmacophore models that included excluded volumes proved to have a better performance. This can be seen in VEGFR2_Ph4_108 and VEGFR2_Ph4_109, which have the same pharmacophoric features but only differ in the number of excluded volumes. As for VEGFR2_Ph4_108, there were 359 excluded volumes added whilst VEGFR2_Ph4_109 had 378 excluded volumes. This difference led to a distinguishable impact on their F1 scores, 0.38 and 0.48, respectively. Adding to that, FAK_Ph4_12 and FAK_Ph4_13 also had the exact same set of features and only varied in the size of the hydrophobic features and number of excluded volumes (68 *vs* 10 excluded volumes, respectively). This difference led to a drastic impact on the model sensitivity and a decrease in the number of false positives obtained by FAK_Ph4_12 versus FAK_Ph4_13 by 159 compounds, which in turn led to a great increase in the overall performance of FAK_Ph4_12 (F1 score of 0.61 for FAK_Ph4_12 vs 0.14 for FAK_Ph4_13).

### Selected 3D pharmacophore models

One of the main assessments and selection criteria of pharmacophore models is their ability to describe and to rationalize the reported experimental structure activity relationship (SAR). Therefore, although model VEGFR2_Ph4_109 having the third best F1 score after VEGFR2_Ph4_103 and VEGFR2_Ph4_104 (Table 3), it was chosen as the best VEGFR2 pharmacophore model as it efficiently describes the previously reported SAR for type II kinase inhibitors (Fig. 4). Likewise, model FAK_Ph4_12, having the third best F1 score after FAK_Ph4_9 and FAK_Ph4_10 (Table 4), was chosen as the best FAK pharmacophore model as it efficiently describes the previously reported SAR for type II kinase inhibitors as well (Fig. 4).

Based on the aforementioned findings, pharmacophore models VEGFR2_Ph4_109 and FAK_Ph4_12 were chosen as the best models amongst the generated models to be progressed to virtual screening.

### VEGFR2_Ph4_109

As can be seen in Table 3, VEGFR2_Ph4_109 chose 38 active compounds out of 40, and 82 decoys out of 1200 as hits. Thus, it showed good sensitivity (Se = 0.95) and specificity (Sp = 0.93). It has a discrimination ratio (DR) of 1.02, showing a balanced tendency to choose true positives and reject true negatives, both of which are in the 90–95% range. It also has a yield of actives (Ya) of 31%, enrichment (E) of 9.82, and accuracy (acc) of 0.93, suggesting that it is far superior to random selection in terms of reliably identifying active hits. VEGFR2_Ph4_109 is the perfect example that fits all the required aspects in the best pharmacophore model, because it is sensitive and specific with a balanced ability in identifying true actives as hits (TP) and discarding inactive compounds (TN).

As only 38 TP were chosen from the 40 actives using Model VEGFR2_Ph4_109, two FN compounds were missed and were not mapped on to the chosen pharmacophore, which can be attributed to the following: (1) they bind to the binding pocket with a different binding pattern other than the training set compounds, or (2) the conformational search algorithm did not yield a conformational combination comprising the conformer that could be mapped onto the pharmacophore of interest.

**Fig. 6:**
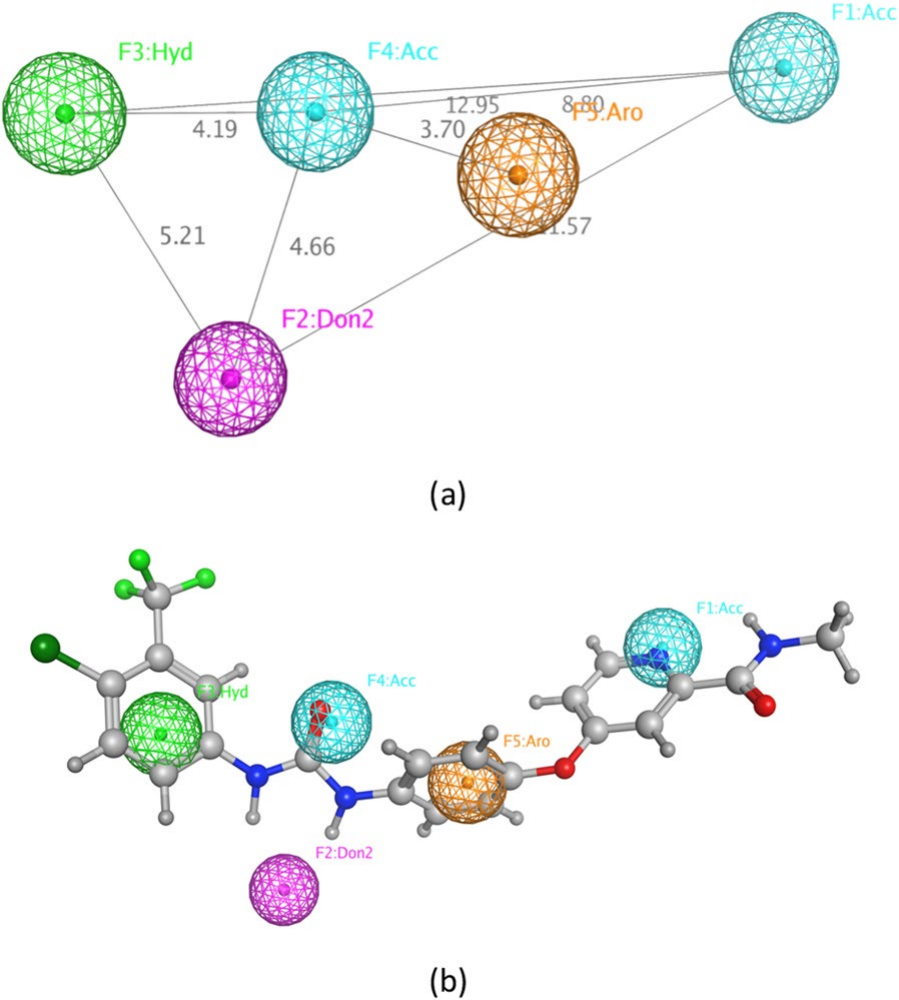
**a** The selected pharmacophore model for VEGFR2 inhibitors, VEGFR2_Ph4_109 (distances in Å) **b** VEGFR2_Ph4_109 mapped onto a VEGFR2 inhibitor

Figure 6a shows the five features of pharmacophore model VEGFR2_Ph4_109 with its inter-feature distances (in Å). First, a hydrogen bond acceptor feature (**F1:Acc**) was used to denote the moiety which interacts with Cys919 at the hinge region. In the gate area, the projection feature (**F2:Don2**) defines the direction of the hydrogen bond donor towards the Glu885 amino acid residue. In addition to the essential hydrophobic feature (**F3:Hyd**) which describes the hydrophobic moiety interacting with the hydrophobic back pocket. Another essential feature is the hydrogen bond acceptor (**F4:Acc**) which represents the moiety that interacts with the key amino acid Asp1046 at the gate area. Lastly, the aromatic scaffold of the inhibitor was described using the aromatic feature (**F5:Aro**). Non-essential features were considered as optional features, meaning that the compounds might or might not have them. 378 excluded volumes were also added to this pharmacophore with the purpose of defining the steric extent of the binding site.

Figure 6b shows the selected pharmacophore model VEGFR2_Ph4_109 mapped onto a representative VEGFR2 inhibitor (Sorafenib) with an RMSD of 0.355 Å from VEGFR2_Ph4_109 feature centers. The low RMSD value demonstrates the fact that the compound's pharmacophoric features are well aligned with the pharmacophore’s feature centers. Its picoline ring satisfies one of the crucial pharmacophoric features for binding in the hinge region, **F1:Acc**, mimicking the ATP nucleotide interactions. The carbonyl oxygen of the urea group is mapped onto **F4:Acc**, representing the essential moiety for interacting with Asp1064. **F2:Don2** lies in position of Glu885 in front of the NHs of the urea group as H-bond donors. The 4-chloro-3-triflourophenyl moiety satisfies the hydrophobic feature **(F3:Hyd)**. Finally, the aromatic ring in the middle of the compound satisfies **F5:Aro**.

### FAK_Ph4_12

As can be seen in Table 4, model FAK_Ph4_12 recognized 15 out of the 17 active compounds (TP = 15) indicating the good model sensitivity (Se = 0.88). Moreover, it exhibited high specificity as well (Sp = 0.98) as it disregarded 966 decoy compounds out of the 983 decoy compounds in the test set (TN = 966). The model recognized a total of 32 compounds as hits of them 15 were active compounds (TP), whereas 17 compounds were inactive (FP) giving a yield of actives (Ya) of 47%. Furthermore, it showed an enrichment value (E) of 27.57 proving the success of the pharmacophore model in improving the selection process of active compounds via the virtual screening technique versus random methods. Moreover, FAK_Ph4_12 model had an accuracy (acc) of 0.98 emphasizing that it can accurately identify active compounds while dismissing the inactive ones. Lastly, it had a discrimination ratio (DR) of 0.90, which shows that this model has a high prediction potential for the inactive compounds compared with the active compounds.

**Fig. 7:**
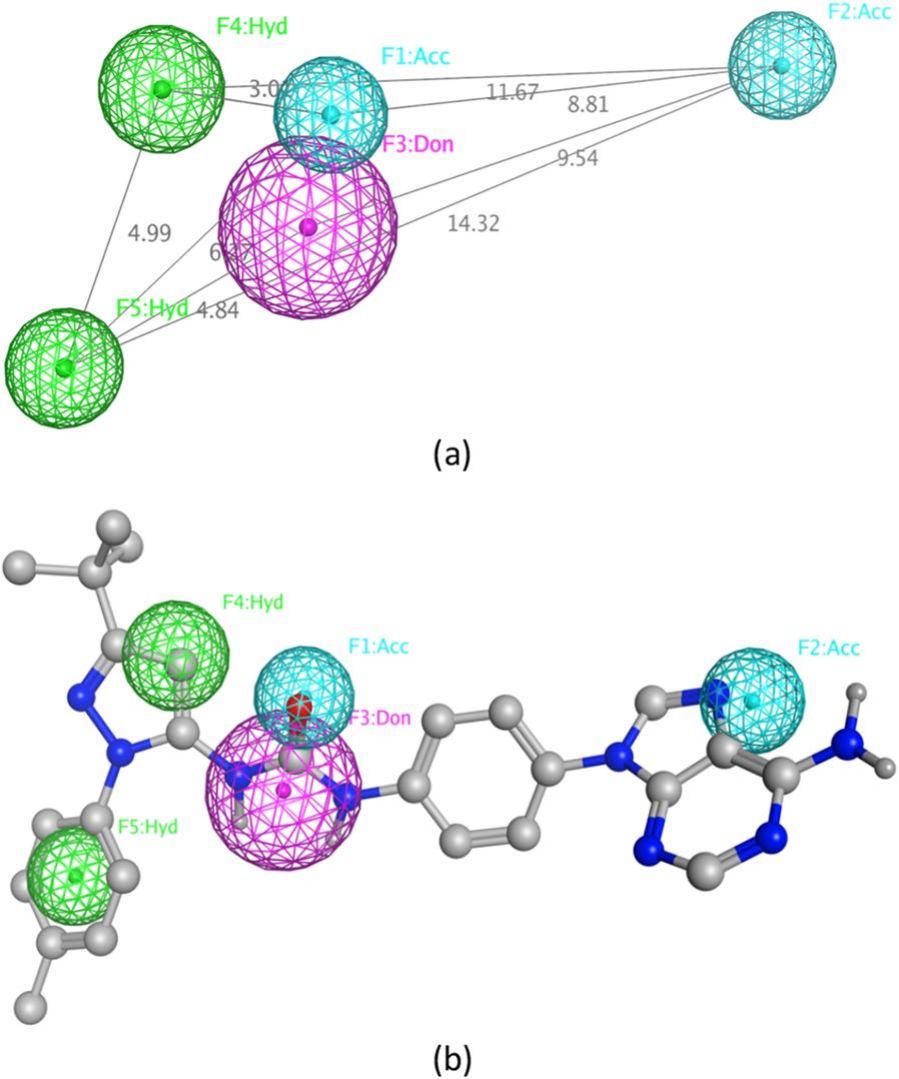
**a** The selected pharmacophore model for FAK inhibitors, FAK_Ph4_12 (distances in Å) **b** FAK_Ph4_12 mapped onto an FAK inhibitor

Figure 7a shows the selected 3D pharmacophore model, FAK_Ph4_12, its pharmacophoric features, and the inter-feature distances (in Å) between each other in 3D space. This pharmacophore model consists of five main features; Feature 1 (**F1:Acc**), a hydrogen bond acceptor, where the ligands bind to Asp564 of the DFG motif at the activation loop. Feature 2 (**F2:Acc**), a hydrogen bond acceptor, maps where the ligands bind to Cys502 residue in the hinge region. Feature 3 (**F3:Don**), a hydrogen bond donor, which describes the feature required for binding to Glu471 residue of the Glu-Lys conserved pair in the αC helix of the N-lobe. Finally, features 4 and 5 (**F4:Hyd** and **F5:Hyd**) where the ligand hydrophobic moieties occupy the allosteric hydrophobic back pocket next to the ATP binding site. Sixty-eight excluded volumes were also added to this pharmacophore with the purpose of defining the steric extent of the binding site.

Figure 7b shows the selected pharmacophore model FAK_Ph4_12 mapped onto a representative FAK inhibitor. The mapped conformer exhibited a low RMSD of 0.419 Å from FAK_Ph4_12 feature centers, demonstrating the good alignment of its pharmacophoric features to FAK_Ph4_12 feature centers. The purine nitrogen is mapped onto pharmacophoric feature **F2:Acc** in the hinge region, the urea group onto features **F1:Acc** and **F3:Don** which lie in the gate area, and lastly the 5-tert-butyl-2-*p*-tolyl-pyrazole ring is mapped onto the two hydrophobic features **F4:Hyd** and **F5:Hyd** residing in the hydrophobic back pocket.

### Virtual screening and hit filtration

In the current research, ZINCPharmer web tool was used to perform the virtual screening step. It is an online interface used for screening the purchasable chemicals from the ZINC database for promising hits searching millions of conformations in just a few minutes [73, 74]. The two selected 3D pharmacophore models for both proteins were used to virtually screen the ZINC database separately and two different sets of hits were obtained, one for VEGFR2 and one for FAK. ZINCPharmer has several filters that can be used to narrow down the retrieved hits. In the current virtual screening, the selection was confined to compounds with molecular weight between 350 and 500 Da and rotatable bonds less than 10 [72].

**Fig. 8:**
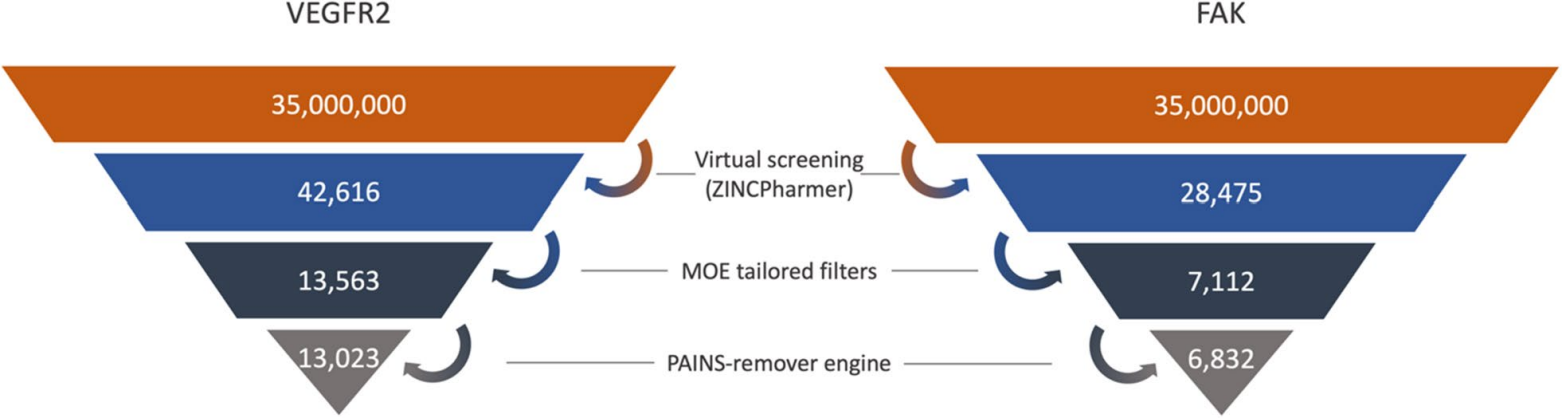
Virtual screening hit filtration for FAK and VEGFR2

ZINCPharmer virtual screening yielded 42,616 hits for VEGFR2 and 28,475 hits for FAK as depicted in Fig. 8. Then, MOE software was used to exclude duplicate compounds MOE. Furthermore, MOE was employed for the selection of lead-like compounds according to various filters (Table 5). These filters include Oprea lead-like filter, compounds that violated more than one criterion of Lipinski's rule of five, mutagenic compounds, and those with a topological polar surface area (TPSA) more than 140 Å^2^, as well as hits with a Log S less than − 5 [75–79]. Furthermore, the PAINS-Remover engine (https://www.cbligand.org/PAINS) was used to ensure that the chosen hits were neither frequent hitters nor promiscuous compounds that could show positive response in assays independent of the protein target and hence have many negative consequences [80]. This filtration process yielded a total of 13,023 compounds for VEGFR2 and 6,832 compounds for FAK (Fig. 8).

**Table 5:** Hit filtration criteria

Criterion	Cutoff
Lipinski’s rule violation count	≤ 1
Molecular weight	< 500
logP	≤ 5
# HBA	≤ 10
# HBD	≤ 5
Veber’s rule	
Number of rotatable bonds	≤ 10
Polar surface area	< 140 Å^2^
LogS	≥ − 5

The final survived hits for both targets were compared against each other to search for common compound in both sets of hits. This resulted in 124 compounds, which could have potential dual VEGFR2/FAK inhibitory activity.

Despite the large size of the screened database, the identified compounds still represent a small portion of the chemical space, and a more extensive exploration of further chemical libraries using the selected pharmacophore models may be necessary to identify further novel dual VEGFR2/FAK kinase inhibitors.

### Molecular docking

Molecular docking simulations were carried out for the 124 common compounds to study their binding pattern and protein–ligand interactions in both the VEGFR2 and FAK binding sites to validate their proposed VEGR2/FAK dual inhibitory activity.

VEGFR2-PDB ID: 4ASD [60] and FAK-PDB ID: 4K9Y [61] were used to perform the molecular docking study. First, self-docking of the co-crystallized ligands in the binding pocket of VEGFR2 and FAK was used to validate the molecular docking protocols to be used. Self-docking gave docking poses with energy scores (S) =  − 15.24 and − 16.02 kcal/mol and RMSD of 0.355 and 0.151 Å from the co-crystalized ligand poses in VEGFR2 and FAK, respectively (For further details, see Additional file 1; S4.1. Self-docking molecular docking validation). Due to its ability to mimic the poses of the co-crystallized ligands and their interactions in the VEGFR2 and FAK binding site, the docking protocol validation step suggested that the used docking protocol was appropriate for carrying out the intended molecular docking studies.

**Fig. 9:**
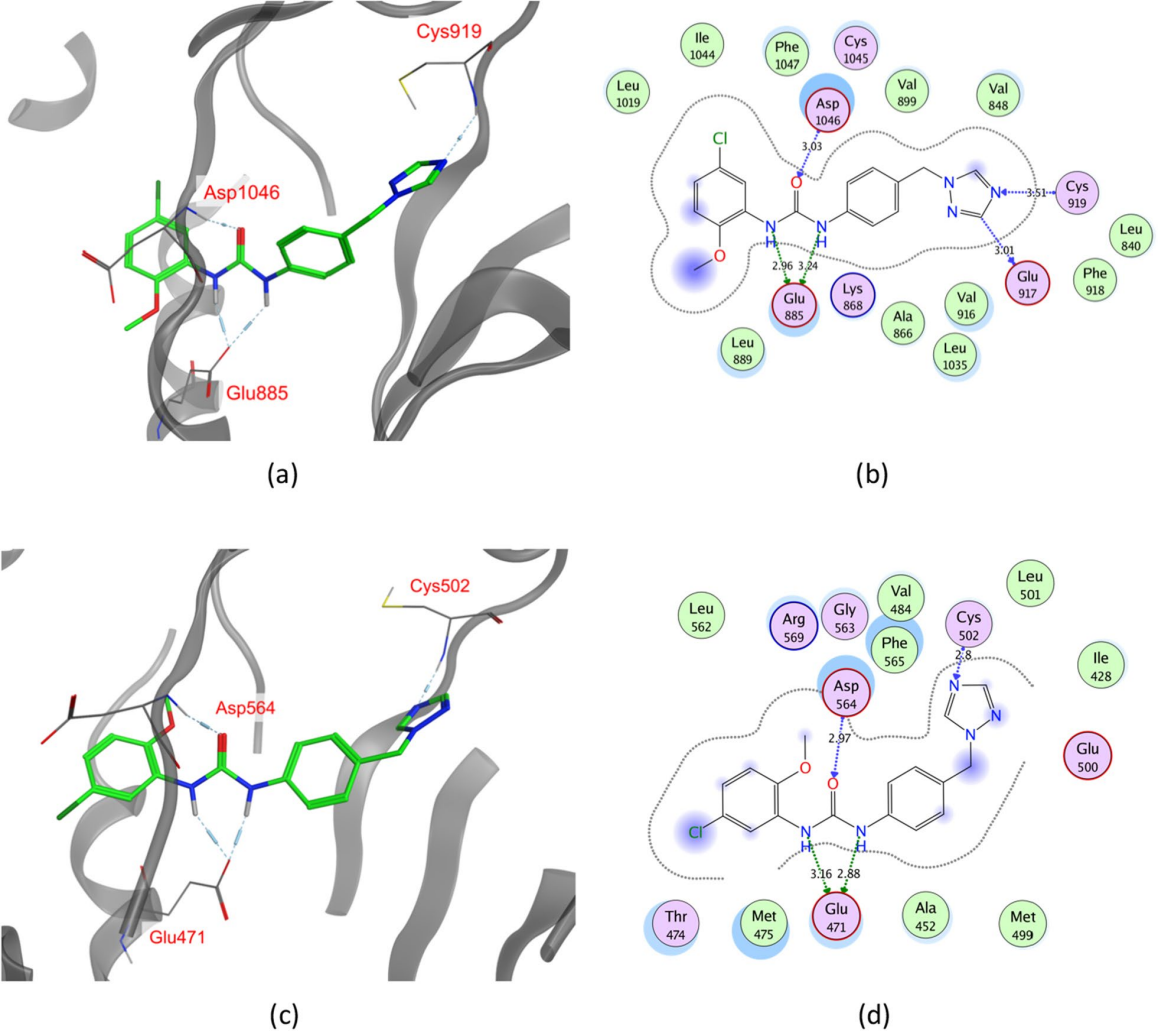
ZINC09875266 docked into FAK and VEGFR binding sites. **a** VEGFR 3D representation. **b** VEGFR2 2D representation. **c** FAK 3D representation. **d** FAK 2D representation

Based on the molecular docking study, out of the 124 compounds, thirteen compounds were found to satisfy all necessary interactions with VEGFR2 and FAK binding site (Hinge region VEGFR2-Cys919 and FAK-Cys502, DFG motif VEGFR2-Asp1046 and FAK-Asp564, αC-helix of gate area VEGFR2-Glu885 and FAK-Glu471) and thus they are expected to have a possible dual binding to both kinases. Figure 9 shows a representative compound for the promising thirteen compounds (ZINC09875266) performing the key interactions in both kinases. This compound’s binding pattern represents that exhibited by the 13 compounds that were able to achieve all essential interactions with the key amino acids in the hinge region and gate area, while extending to the hydrophobic back pocket of the binding site. See additional file 1; S4.2. Docking energy score (S) in kcal/mol for the common 13 compounds in VEGFR2 and FAK binding sites, for the ZINC ID of the 13 promising compounds with their predicted binding scores in the kinase domains of the target proteins VEGFR2 and FAK.

### Pharmacokinetic properties prediction

**Fig. 10:**
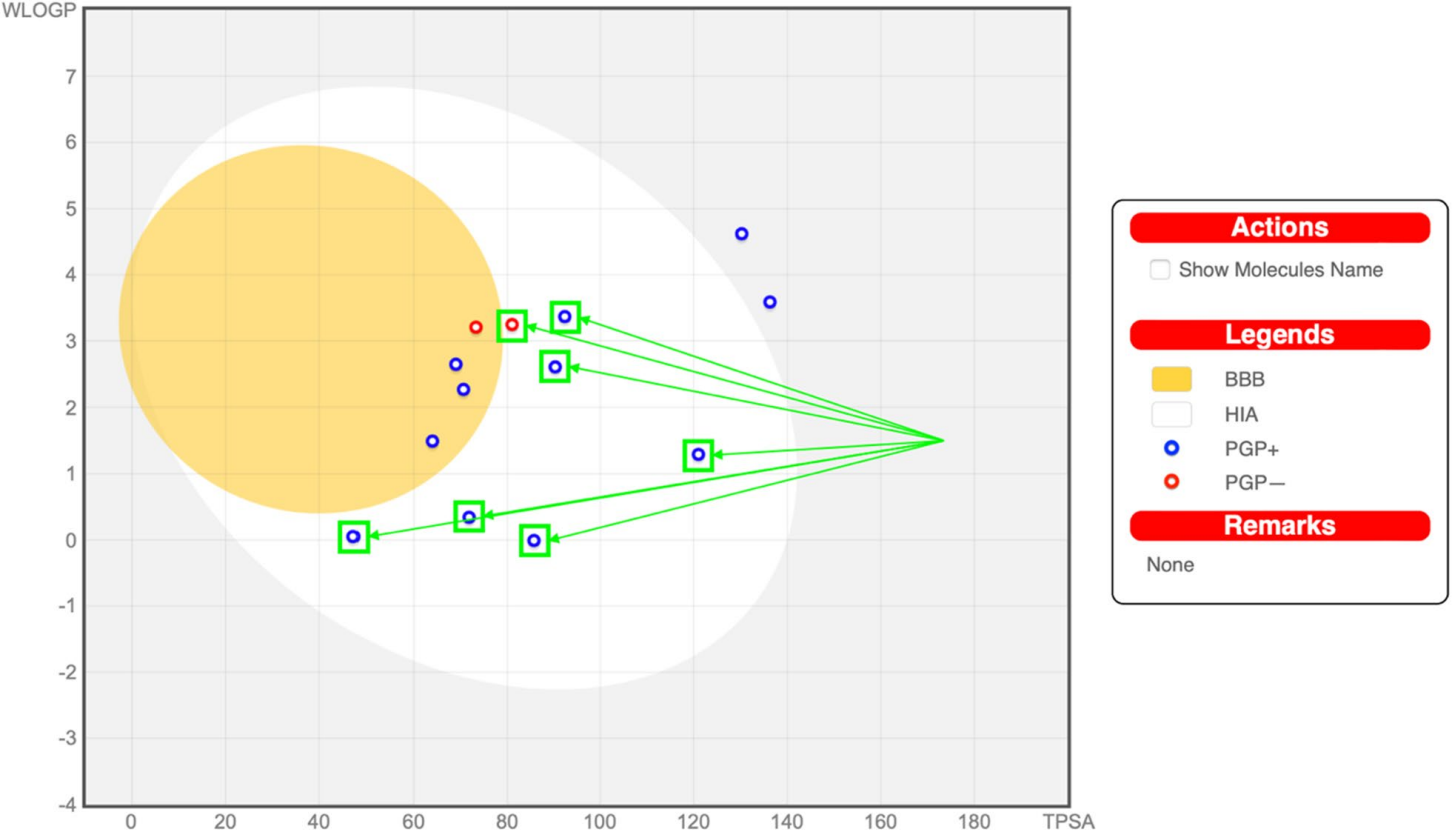
SwissADME Boiled-Egg plot dividing the compounds into three regions: bad oral bioavailability (grey), good oral bioavailability (white), and BBB permeation (yellow). The highlighted points represent the compounds with desirable characters

**Fig. 11:**
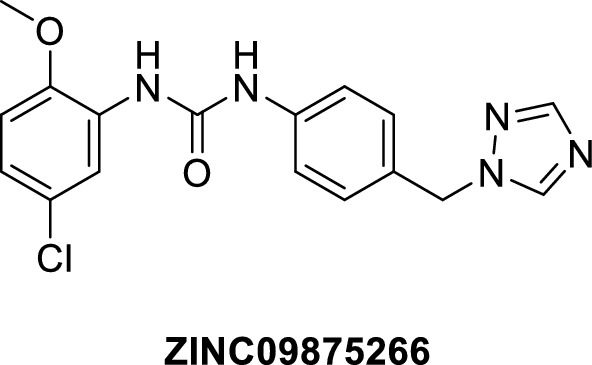
The chemical structure of the hit compound survived the pharmacokinetics filtration step

The thirteen common hit compounds showing promising binding pattern in the binding sites of the target kinases were subjected further to assessment of their pharmacokinetic properties. This step is crucial to ensure that the chosen hit compounds do not only have good binding patterns to our targets, but also have desirable ADME characters, meaning they are likely to reach their targets in sufficient concentrations, resist degradation in biological environments for a reasonable duration of action, and with limited side effects. This was done using SwissADME web tool (http://www.swissadme.ch) [81]. Figure 10 shows the obtained SwissADME Boiled‒Egg plot, which predicts both the GIT absorption and BBB permeation of the examined compounds [81, 82]. The prediction depends on two physicochemical parameters, wlogP and topological polar surface area (TPSA) [82]. The white region is the physicochemical space of molecules with highest probability of GIT absorption, and the yellow region (yolk) is the physicochemical space of molecules with highest probability of BBB permeation. Compounds predicted to be P-glycoprotein (P-gp) substrates are shown in blue, whereas compounds in red are not. Seven compounds were predicted to be GIT absorbable without BBB permeation (i.e., no central side effects), from which only one P-glycoprotein (P-gp) non-substrate; compound ZINC09875266 (Fig. 11). It also showed a promising synthetic accessibility of 2.42 according to SwissADME prediction, in a range of 1 to 10 where 1 is very easy and 10 is very difficult to synthesize.

These results indicate that compound ZINC09875266 (Fig. 11) is not only promising in terms of binding patterns to our target kinases, but also in terms of its pharmacokinetic properties. Noteworthy, these findings are computational prediction and further in vitro and in vivo experimental studies are required to validate the potential of the identified compounds as novel cancer treatments.

## Conclusion

In conclusion, this study focused on the discovery of hit compounds that could act as dual-kinase inhibitors targeting VEGFR2 and FAK for potential application in cancer treatment. Through receptor-based pharmacophore modeling, a set of compounds with promising predicted binding capability against both targets was identified. a lead-like compound (ZINC09875266) was selected as a potential candidate for further exploration in designing novel dual-kinase inhibitors. The outlook of the current work is that the most promising molecules are to be tested in vitro on VEGFR2 and FAK enzymes and on cancer cells and in vivo using cancer animal models.

## Methodology

Unless otherwise stated, all molecular modelling studies were carried out using Molecular Operating Environment (MOE, 2020.0901) software.

### Protein structure similarity assessment

NCBI Basic Local Alignment Search Tool (BLAST) (https://blast.ncbi.nlm.nih.gov/Blast.cgi) was used to carry out the similarity search between the kinase domains of VEGFR2 and FAK. BLAST finds regions of local similarity between sequences. The protein BLAST (BLASTp) was chosen for identifying similarity in amino acid sequences of both proteins. First, the Protein Data Bank (https://www.rcsb.org) was used to get the amino acid sequence of both proteins’ kinase domains in FASTA format (VEGFR2-PDB-ID: 4ASD, FAK-PDB-ID: 4K9Y) [60, 61]. These protein sequences were chosen on the basis of being wild type and non-mutant. The VEGFR2 kinase domain sequence was input as the query sequence, while the FAK kinase domain sequence was input as the subject sequence, then multiple sequence alignment of the amino acids was carried out.

### Retrieving X-ray crystallographic structures and training set generation

After searching the protein data bank for crystallographic structures of the proteins co-crystalized with type II inhibitors, we found seventy-four structures for VEGFR and only two structures for FAK. For our training set, we selected ten X-ray crystallographic structures of the proteins co-crystalized with type II inhibitors, eight structures for the VEGFR2 (Table 1) and two structures for the FAK (Table 2).

The X-ray crystallographic structures of VEGFR2 and FAK co-crystallized with different type II inhibitors (VEGFR2-PDB ID: 4ASE, 4ASD, 2QU6, 3VHE, 3EWH, 3VNT, 3WZD, and 6XVK. FAK-PDB ID: 4KAO and 4K9Y) [47, 60, 61, 67–71] were downloaded from the Protein Data Bank (https://www.rcsb.org/). All the downloaded co-crystal structures were inspected, and it was confirmed that they perform interactions with all key amino acid residues; VEGFR2-Asp1046 and FAK-Asp564 of the conserved DFG-motif, VEGFR2-Glu885 and FAK-Glu471 of the αC-helix at the gate area, and lastly VEGFR2-Cys919 and FAK-Cys502 of the hinge region.

Molecular Operating Environment (MOE, 2020.0901) software was used to prepare the proteins. Chain A was kept in all protein structure, other chains (if any), water molecules (if any) and superfluous ligand molecules that are not involved in the ligand-target interactions were removed. The protein structures were then prepared using *QuickPrep* protocol in MOE with default options.

### Pharmacophore model generation

The prepared protein structures from the PDB for VEGFR2 and FAK (VEGFR2-PDB ID: 4ASE, 4ASD, 2QU6, 3VHE, 3EWH, 3VNT, 3WZD, and 6XVK. FAK-PDB ID: 4KAO and 4K9Y) [47, 60, 61, 67–71] containing the co-crystallized inhibitors were aligned separately, then superposed using *Align* protocol in MOE using protein structures’ αCs.

Using *pharmacophore query editor* in MOE, the aligned ligands were used to generate several manual 3D pharmacophore models for each set of aligned structures based on their common interactions with the target kinase binding site. The main common ligand-target interactions include H-bonding interactions with the hinge region VEGFR2-Cys919 and FAK- Cys502, DFG VEGFR2-Asp1046 and FAK-Asp564, αC-helix VEGFR2-Glu885 and FAK-Glu471, in addition to hydrophobic interactions with hydrophobic side chains of the amino acids lining the hydrophobic allosteric back pocket [25, 44, 61, 64, 65]. Moreover, several excluded volumes (with different volumes and number) were included to define the steric extent of the binding site.

### Test set compilation

The ultimate goal in virtual screening is to use a protocol that is sensitive in filtering the maximum number of active hits and simultaneously specific in screening out almost all inactive compounds. Thus, in the current work, a test set of active inhibitors and decoys was constructed for each protein kinase to test and validate the different manually generated pharmacophore models to select the best performing pharmacophore model in discriminating between active compounds and decoys for each protein kinase.

This test set contains 2240 compounds, including 1240 compounds for VEGFR2 and 1000 compounds for the FAK. The VEGFR2 test set comprised 39 active compounds (see Additional file 1: Table S1. VEGFR2 test set active compounds) and 1200 decoy compounds, obtained from DEKOIS 2.0 database of benchmark data set (www.dekois.com). Whilst the FAK test set contains 45 self-collected compounds, which included 17 active compounds and 28 inactive compounds (see Additional file 1: Table S2. FAK manually collected test set compounds) [61, 83–87], as well as 955 decoys retrieved from the DUD-E decoy generator [88] (http://dude.docking.org/generate). Activity status was determined based on their biochemical IC_50_ values, with a cutoff of 10 µM (active < 10 µM < inactive) as conventionally known for kinase inhibitors [89].

The compiled test set compounds were exposed to MOE database *Wash* module which defines the most probable protonation state of strong acids and strong bases in aqueous near-neutral environment to be used. For functional groups which have a pKa close to 7, which cannot reasonably be classified as being exclusively protonated or deprotonated, the protonation state of the unionized input molecule is used. Then energy minimized until an RMS gradient of 0.1 kcal mol^−1^ Å^−2^ using MOE with Amber10:EHT force field. Conformational search was then carried out using *LowModeMD* method in MOE, this method is intended for large, perhaps disconnected, complex structures like macrocycles and protein loops, but it can also be employed for detailed, accurate analysis of small molecules [90]. It generates conformations by utilizing short ≈ 1 ps runs of molecular dynamics at a constant temperature then performs an all-atom energy minimization. This resulted in 43,038 conformers for the VEGFR2 test set compounds and 33,362 conformers for the FAK test set compounds, which were then used for pharmacophore model selection and validation.

### Pharmacophore selection and validation

Using MOE *Pharmacophore Search* module, the generated test set conformers were screened using the different manually generated structure-based pharmacophore models to test their ability to discriminate between the active and decoy compounds of the compiled test sets. MOE pharmacophore search-algorithm starts by prefiltering the conformers based on the feature types and distance similarity to the ones mapped on the pharmacophore model; followed by a more expensive alignment of the conformer atoms to the query feature points minimizing their deviation from each other. The quality of the alignments is determined using root mean square deviation (RMSD) as a fitness criterion.

Various assessment metrics were utilized to assess the performance of the different generated pharmacophore models to select and validate the best one for each protein kinase. The screening output of the test sets [True positive (**TP**), true negative (**TN**), false positive (**FP**), and false negative (**FN**)] was used to calculate these assessment metrics. These metrics include sensitivity (**Se**), specificity (**Sp**), yield of actives (**Ya**), enrichment (**E**), accuracy (**acc**), discrimination ratio (**DR**), and F1 score (**F1**) (for further details see Additional file 1: Table S3. Assessment metrics of pharmacophore models performance).

### Virtual screening

According to the previously mentioned assessment metrics, the best performing pharmacophore models (best discrimination between actives and decoy compounds) for VEGFR2 and FAK were used to virtually screen the ZINC database (purchasable subset) to obtain two separate sets of hits for VEGFR2 and FAK [73]. This was done using ZINCPharmer web tool [74], a free online virtual screening tool which screens the ZINC database (http://zincpharmer.csb.pitt.edu). A few filters were used to limit the hits retrieved to those with molecular weight in the range of 350–500 Da and the number of rotatable bonds should not exceed ten [72].

### Hit filtration

The retrieved hits for each kinase from the virtual screening step were then subjected to several consecutive filtration stages. Initially, the duplicate hits were removed using MOE *unique* molecule selection. The remaining hits were then filtered using the following filters (Table 5); mutagenic compounds were identified and removed based on the work of Kazius et al*.* [75]*,* using MOE. Furthermore, using MOE, the Oprea lead-like filter was then applied to select only compounds possessing lead-like properties [76]. The remaining compounds were then subjected to further filtration to keep those comply with Lipinski’s rule of 5 [77], Veber’s rule [78], and a logS value range of lead-like compounds [79] (Table 5).

Finally, Pan-Assay Interference compounds (PAINS)-containing hits were removed using the online PAINS removal tool (https://www.cbligand.org/PAINS) [80]. These compounds are expected to be promiscuous and frequent hitters which can bind to many endogenous targets leading to several off-target side effects [91].

The two sets of hits were compared, and common compounds were identified using MOE software and they were carried forward for the molecular docking studies.

### Molecular docking

The previously prepared VEGFR2 X-ray crystallographic structure PDB ID: 4ASD was chosen for the molecular docking studies for VEGFR2 hits because its co-crystallized ligand is a well-known potent angiokinase inhibitor (Sorafenib) with an IC_50_ of 2.3 nM [60]. As for the FAK X-ray crystallographic structure PDB ID: 4K9Y was chosen from the two available structures to perform the molecular docking simulations due to its non-mutant structure [61].

Validation of the molecular docking protocol was initially carried out by self-docking of the co-crystallized ligands in the vicinity of VEGFR2 and FAK binding sites, and thus the co-crystallized ligand was utilized to assign the active sites for the molecular docking. Using *Rigid receptor protocol* method, *Triangle matcher* placement method was applied to generate docking poses by aligning ligand atom triplets on triplets of receptor site points. *London dG* scoring function was utilized to evaluate the binding free energy of the ligand in a certain pose within the kinases’ binding sites. This specific scoring function considers the rotational and translational entropy change, molecular flexibility, hydrogen bonding energy and geometry, and the atomic desolvation energy. Self-docking gave docking poses with energy scores (S) =  − 15.24 and − 16.02 kcal/mol and RMSD of 0.355 and 0.151 Å from the co-crystalized ligand poses of VEGFR2 and FAK, respectively. Moreover, they reproduced the key interactions performed by the co-crystalized ligands within the binding sites of the target kinases (See additional file1 for further details; S4.1. Self-docking molecular docking validation).

The validated molecular docking protocol was then used to perform the molecular docking simulations for the common hits to study their binding pattern and protein–ligand interactions in VEGFR2 and FAK binding sites to validate their proposed dual inhibitory effect.

### Pharmacokinetic properties prediction

The pharmacokinetic properties of the promising common compounds were then calculated using SwissADME web tool (http://www.swissadme.ch) [81]. SwissADME evaluates drug-likeness, pharmacokinetic characteristics, and medicinal chemistry friendliness of small drug-like molecules [81]. This was performed to ensure that the discovered hit molecules are not only with promising VEGFR2/FAK binding capabilities but also promising ADME properties. Molecules were filtered according to several criteria. Molecules with predicted low gastrointestinal absorption, predicted potential blood brain barrier permeation, or predicted to be P-glycoprotein substrates were removed.

### Supplementary Information

**Additional file 1: S1.** Training set compounds for VEGFR2 and FAK pharmacophore model generation. **S2.** Test set compilation. **Table S1.** VEGFR2 test set active compounds. **Table S2.** FAK manually collected test set compounds. **S3.** Pharmacophore model selection and validation. **Table S3.** Assessment metrics of pharmacophore models performance. **Table S4.** VEGFR2 pharmacophore model assessment. **S4.** Molecular docking simulation. S4.1. Self-docking molecular docking validation. S4.1.1. Self-docking validation for VEGFR2. S4.1.2. Self-docking validation for FAK. S4.2. Docking energy score (S) in kcal/mol for the common 13 compounds in VEGFR2 and FAK binding sites.

## Data Availability

All data generated or analyzed during this study are included in this published article and its supplementary information files.
