# Supplementary material for: Discovery of dual kinase inhibitors targeting VEGFR2 and FAK: structure-based pharmacophore modeling, virtual screening, and molecular docking studies
Source: BMC Chem. 2024 Feb 12;18(1):29. doi: 10.1186/s13065-024-01130-5 (PMC10863211; doi:10.1186/s13065-024-01130-5)
Supplement: Supplementary file 1 — Additional file 1: S1. Training set compounds for VEGFR2 and FAK pharmacophore model generation. S2. Test set compilation. Table S1. VEGFR2 test set active compounds. Table S2. FAK manually collected test set compounds. S3. Pharmacophore model selection and validation. Table S3. Assessment metrics of pharmacophore models performance. Table S4. VEGFR2 pharmacophore model assessment. S4. Molecular docking simulation. S4.1. Self-docking molecular docking validation. S4.1.1. Self-docking validation for VEGFR2. S4.1.2. Self-docking validation for FAK. S4.2. Docking energy score (S) in kcal/mol for the common 13 compounds in VEGFR2 and FAK binding sites. [file 13065_2024_1130_MOESM1_ESM.docx]

Additional file for

Discovery of Dual Kinase Inhibitors Targeting VEGFR2 and FAK: Structure-based Pharmacophore Modeling, Virtual Screening, and Molecular Docking Studies

Marwa A. Fouad^a,b^, Alaa A. Osman^b^, Noha M. Abdelhamid^b^, Mai W. Rashad^b^, Ashrakat Y. Nabawi^b^, Ahmed M. El Kerdawy^a,b,c^

*^a^ Pharmaceutical Chemistry Department, Faculty of Pharmacy, Cairo University, Kasr El-Aini St., Cairo 11562, Egypt*

*^b^ Pharmaceutical Chemistry Department, School of Pharmacy, Newgiza University (NGU), Newgiza, km 22 Cairo-Alexandria Desert Road, Cairo, Egypt.*

*^c^ School of Pharmacy, College of Health and Science, University of Lincoln, Joseph Banks Laboratories, Green Lane, Lincoln, Lincolnshire, United Kingdom.*

***Corresponding author:*** Marwa Fouad, [marwa.fouad@pharma.cu.edu.eg](mailto:marwa.fouad@pharma.cu.edu.eg)

**Contents**

**S1.** Training set compounds for VEGFR2 and FAK pharmacophore model generation.

**S2.** Test set compilation

Table S1. VEGFR2 test set active compounds.

Table S2. FAK manually collected test set compounds.

**S3.** Pharmacophore model selection and validation

Table S3. Assessment metrics of pharmacophore models performance.

Table S4. VEGFR2 pharmacophore model assessment

**S4.** Molecular docking simulation

S4.1. Self-docking molecular docking validation

S4.1.1. Self-docking validation for VEGFR2

S4.1.2. Self-docking validation for FAK

S4.2. Docking energy score (S) in kcal/mol for the common 13 compounds in VEGFR2 and FAK binding sites

**References**

**S1. Training set compounds for VEGFR2 and FAK pharmacophore model generation**

**Compound VEGFR_1** – Tivozanib has a high affinity to VEGFR-2 with an IC_50_ value of 0.04 nM. It binds to VEGFR-2 by forming hydrogen bonds at the hinge region, gate area, and the hydrophobic back pocket. In the gate area, its carbonyl group forms a hydrogen bond with the amino acid residue Asp 1046, while its crucial urea group forms another hydrogen bond with the conserved Glu885 of αC helix. On the other hand, the quinoline nitrogen interacts through hydrogen bonding with Cys919. [1]

**Compound VEGFR_2** – Sorafenib forms various hydrogen bonds with the key amino acid residues. With an IC_50_ value of 2.3 nM, this compound shows a high affinity towards VEGFR-2. Both the carboxamide nitrogen and the pyridine moiety are accommodated and interact through hydrogen bonding with the amino acid residue Cys919 at the front cleft (hinge region). Also, in the gate area, **compound 2** hydrogen bonds *via* its urea nitrogen groups with Glu885 of αC helix, on the other hand, the carbonyl group performs additional hydrogen bond interactions with Cys1045 and Asp1046. Whilst the 1-chloro-2-trifluoromethyl-benzene moiety occupies the hydrophobic back pocket. [1]

**Compound VEGFR_3** – It has an affinity to VEGFR-2 with an IC_50_ value of 15 nM. Both nitrogen atoms present in the pyridine ring and the carboxamide group perform hydrogen bonding with the Cys919 amino acid residue of the hinge region. **Compound 3** extends beyond gatekeeper Val916, consequently, the benzoxazole core is accommodated in the hydrophobic pocket. Moreover, the endocyclic nitrogen of the aminobenzoxazole hydrogen bonds with Asp1046 and the exocyclic NH performs a hydrogen bond interaction with the side chain of Glu885 of αC helix.[2]

**Compound VEGFR_4** – It interacts *via* hydrogen bond formation with the binding pocket. It has an affinity towards VEGFR-2 with an IC_50_ value of 6.2 nM. N1-nitrogen of the pyrrolo[3,2- d]pyrimidine core interacts with Cys919 in the hinge region *via* hydrogen bond formation. Both urea groups form hydrogen bonds with Glu885 of αC helix, and the carbonyl group performs an additional hydrogen bond interaction with Asp1046 in the conserved DFG motif. Moreover, 3-(trifluoromethyl) phenyl moiety is accommodated in the hydrophobic back pocket generated by DFG-out conformation.[3]

**Compound VEGFR_5** – It has an affinity towards VEGFR-2 with an IC_50_ value of 69±10 nM. The aminobenzimidazole occupies the gate area, with HB interactions with α-C-helix residue Glu885 and the backbone N–H of activation loop residue Asp1046**.** The methyl-aminopyrimidine moiety is accommodated and hydrogen bonds with the Cys919 at the hinge region. Additionally, a striking increase in potency is achieved by the trifluoromethyl-arene portion of the benzimidazole which is accommodated at the back hydrophobic pocket.[4]

**Compound VEGFR_6** – It interacts with VEGFR-2 *via* hydrogen bonding and shows high affinity with an IC_50_ value of 2.2 nM. The carbonyl oxygen interacts with the Asp1046 *via* hydrogen bond interaction. Additionally, the carboxamide group forms a hydrogen bond with α-C-helix residue Glu885. While in the hinge region, the nitrogen of the thiazole ring interacts with Cys919 *via* hydrogen bond interaction. 3-(1-cyanocyclopropyl) benzene occupies the hydrophobic back pocket generated by DFG-out conformation.[5]

**Compound VEGFR_7** – **Lenvatinib** has an IC_50_ value of 5.1 nM, which reveals a high inhibitory effect against VEGFR-2[6]. In the gate area, the carbonyl oxygen interacts with the Asp1046 of the conserved DFG motif *via* a hydrogen bond, and the urea groups interact through hydrogen bonding with Glu 885 of α-C-helix. At the hinge region, the quinoline ring is accommodated and interacts by its nitrogen atom with Cys919 through hydrogen bonding. While the cyclopropane moiety occupies the hydrophobic pocket generated by DFG-out conformation.[7]

**Compound VEGFR_8** – It has an affinity towards VEGFR-2 with an IC_50_ value of 0.538 nM. Importantly, in the gate area, the carbonyl-oxygen hydrogen bonds with Asp1046 of the conserved DFG motif and carboxamide NH forms another hydrogen bond with Glu885. Whilst in the hinge region, the quinoline ring is accommodated and performs a hydrogen bond interaction *via* its nitrogen atom with Cys919. Whilst the back pocket is occupied by the benzoxazine moiety.[8]

**Compound FAK_1** – It was discovered during an HTS campaign for FAK inhibitor discovery. It inhibits FAK at a relatively high potency with an IC_50_ of 266 nM. In FAK kinase domain, its urea moiety binds to the gate area via H-bonds with Glu471 and Asp564 as well as to Gly563 preceding the DFG loop. Moreover, the purine scaffold binds to the hinge region by its 5-membered ring by classical and non-classical H-bonding to Cys502 and Glu500, respectively, and with by its exocyclic NH_2_ to Cys502 as well. Binding of this compound to FAK induces conformational shift of Phe565 from the DFG loop toward the ATP binding site (DFG out conformation), exposing the allosteric hydrophobic back pocket, in which the 5-tert-butyl-2-*p*-tolyl-pyrazole ring is embedded.[9]

**Compound FAK_2** – It is the phenyl-urea substituted pyrazole analogue of compound FAK_9 with a six membered ring as a hinge binder. It has a lower affinity to FAK (IC_50_ of 7000 nM) but with greater selectivity. It binds to FAK in a similar manner to compound FAK_9, however, the Cys502 of the hinge region is bound to the pyridine nitrogen via a hydrogen bond**.** This change led to its lower affinity relative to compound FAK_9. Unlike its previous analogue, compound FAK_10 exhibited no cellular activity which indicates the need for its optimization for other factors such as cell permeability, potency, and plasma protein binding.[9]

**S2. Test set compilation**

Table S1. VEGFR2 test set active compounds.

| **ID** | **Structure** | **ID** | **Structure** |
| --- | --- | --- | --- |
| VEGFR_9 |  | VEGFR_10 |  |
| VEGFR_11 |  | VEGFR_12 |  |
| VEGFR_13 |  | VEGFR_14 |  |
| VEGFR_15 |  | VEGFR_16 |  |
| VEGFR_17 |  | VEGFR_18 |  |
| VEGFR_19 |  | VEGFR_20 |  |
| VEGFR_21 |  | VEGFR_22 |  |
| VEGFR_23 |  | VEGFR_24 |  |
| VEGFR_25 |  | VEGFR_26 |  |
| VEGFR_27 |  | VEGFR_28 |  |
| VEGFR_29 |  | VEGFR_30 |  |
| VEGFR_31 |  | VEGFR_32 |  |
| VEGFR_33 |  | VEGFR_34 |  |
| VEGFR_35 |  | VEGFR_36 |  |
| VEGFR_37 |  | VEGFR_38 |  |
| VEGFR_39 |  | VEGFR_40 |  |
| VEGFR_41 |  | VEGFR_42 |  |
| VEGFR_43 |  | VEGFR_44 |  |
| VEGFR_45 |  | VEGFR_46 |  |
| VEGFR_47 |  |  |  |

Table S2. FAK manually collected test set compounds.

| **ID** | **Structure** | **Ref** | **ID** | **Structure** | **Ref** |
| --- | --- | --- | --- | --- | --- |
| FAK_1^a^ |  | [9] | FAK_2^a^ |  | [9] |
| FAK_3^a^ |  | [9] | FAK_4^a^ |  | [9] |
| FAK_5^a^ |  | [9] | FAK_6^a^ |  | [9] |
| FAK_7^a^ |  | [10] | FAK_8^a^ |  | [10] |
| FAK_9^a^ |  | [10] | FAK_10^a^ |  | [10] |
| FAK_11^a^ |  | [11] | FAK_12^a^ |  | [11] |
| FAK_13^a^ |  | [11] | FAK_14^a^ |  | [11] |
| FAK_15^a^ |  | [12] | FAK_16^a^ |  | [12] |
| FAK_17^a^ |  | [12] | FAK_18^i^ |  | [9] |
| FAK_19^i^ |  | [9] | FAK_20^i^ |  | [9] |
| FAK_21^i^ |  | [9] | FAK_22^i^ |  | [9] |
| FAK_23^i^ |  | [9] | FAK_24^i^ |  | [9] |
| FAK_25^i^ |  | [9] | FAK_26^i^ |  | [9] |
| FAK_27^i^ |  | [9] | FAK_28^i^ |  | [9] |
| FAK_29^i^ |  | [9] | FAK_30^i^ |  | [11] |
| FAK_31^i^ |  | [11] | FAK_32^i^ |  | [11] |
| FAK_33^i^ |  | [11] | FAK_34^i^ |  | [13] |
| FAK_35^i^ |  | [12] | FAK_36^i^ |  | [12] |
| FAK_37^i^ |  | [12] | FAK_38^i^ |  | [12] |
| FAK_39^i^ |  | [12] | FAK_40^i^ |  | [12] |
| FAK_41^i^ |  | [12] | FAK_42^i^ |  | [12] |
| FAK_43^i^ |  | [12] | FAK_44^i^ |  | [12] |
| FAK_45^i^ |  | [14] |  |  |  |

^a^ active compound.

^i^ inactive compound.

**S3. Pharmacophore model selection and validation**

Table S3. Assessment metrics of pharmacophore models performance. [15]

| **Assessment metric** | **Equation** | **Insight** |
| --- | --- | --- |
| **Sensitivity (*Se*)** | $Se= \frac{TP}{A}$ | Model ability to select correct active molecules |
| **Specificity (*Sp*)** | $Sp= \frac{TN}{N-A}$ | The goodness of the model in discarding  inactive compounds |
| **Yield of actives (*Ya*)** | $Ya= \frac{TP}{n}$ | The hit rate |
| **Enrichment (*E*)** | $E= \frac{TP/n}{A/N}$ | How many times the virtual screening workflow performs better than a random selection in retrieving active compounds |
| **Accuracy (*acc*)** | $acc= \frac{TP+TN}{N}$ | The accuracy of the virtual  screening workflow in discriminating between active and inactive compounds |
| **Discrimination ratio (*DR*)** | $DR= \frac{Se}{Sp}$ | The ratio in the prediction accuracy of the active and inactive compounds of the virtual screening workflow |
| **F1 Score (*F1*)** | $F1= \frac{TP}{TP+\frac{1}{2}(FP+FN)}$ | Measures the overall quality of the pharmacophore model in discriminating between active and inactive compounds (0 – 1) |

**TP** is the number of true positive, **TN** is the number of true negative, **FP** is the number of false positive, **FN** is the number of false negative, **A** is the number of actives, **n** is the number of selected compounds as hits and **N** is the total number of compounds in the dataset.

Table S4. VEGFR2 pharmacophore model assessment

| Ph4-No. | n | TP | FP | TN | FN | Se | Sp | Ya | E | acc | DR | F1 |
| --- | --- | --- | --- | --- | --- | --- | --- | --- | --- | --- | --- | --- |
| Ph4-1 | 41 | 0 | 1 | 1199 | 40 | 0.00 | 1.00 | 0.00 | 0.00 | 0.97 | 0.00 | 0.00 |
| Ph4-2 | 42 | 1 | 2 | 1198 | 39 | 0.03 | 1.00 | 0.02 | 0.74 | 0.97 | 0.03 | 0.05 |
| Ph4-3 | 113 | 4 | 73 | 1127 | 36 | 0.10 | 0.94 | 0.04 | 1.10 | 0.91 | 0.11 | 0.07 |
| Ph4-4 | 79 | 7 | 39 | 1161 | 33 | 0.18 | 0.97 | 0.09 | 2.75 | 0.94 | 0.18 | 0.16 |
| Ph4-5 | 228 | 13 | 188 | 1012 | 27 | 0.33 | 0.84 | 0.06 | 1.77 | 0.83 | 0.39 | 0.11 |
| Ph4-6 | 64 | 7 | 24 | 1176 | 33 | 0.18 | 0.98 | 0.11 | 3.39 | 0.95 | 0.18 | 0.20 |
| Ph4-7 | 515 | 21 | 475 | 725 | 19 | 0.53 | 0.60 | 0.04 | 1.26 | 0.60 | 0.87 | 0.08 |
| Ph4-8 | 150 | 8 | 110 | 1090 | 32 | 0.20 | 0.91 | 0.05 | 1.65 | 0.89 | 0.22 | 0.10 |
| Ph4-9 | 44 | 2 | 4 | 1196 | 38 | 0.05 | 1.00 | 0.05 | 1.41 | 0.97 | 0.05 | 0.09 |
| Ph4-10 | 59 | 6 | 19 | 1181 | 34 | 0.15 | 0.98 | 0.10 | 3.15 | 0.96 | 0.15 | 0.18 |
| Ph4-11 | 223 | 28 | 183 | 1017 | 12 | 0.70 | 0.85 | 0.13 | 3.89 | 0.84 | 0.83 | 0.22 |
| Ph4-12 | 63 | 16 | 23 | 1177 | 24 | 0.40 | 0.98 | 0.25 | 7.87 | 0.96 | 0.41 | 0.41 |
| Ph4-13 | 600 | 21 | 560 | 640 | 19 | 0.53 | 0.53 | 0.04 | 1.09 | 0.53 | 0.98 | 0.07 |
| Ph4-14 | 427 | 12 | 387 | 813 | 28 | 0.30 | 0.68 | 0.03 | 0.87 | 0.67 | 0.44 | 0.05 |
| Ph4-15 | 50 | 2 | 10 | 1190 | 38 | 0.05 | 0.99 | 0.04 | 1.24 | 0.96 | 0.05 | 0.08 |
| Ph4-16 | 62 | 3 | 22 | 1178 | 37 | 0.08 | 0.98 | 0.05 | 1.50 | 0.95 | 0.08 | 0.09 |
| Ph4-17 | 44 | 3 | 4 | 1196 | 37 | 0.08 | 1.00 | 0.07 | 2.11 | 0.97 | 0.08 | 0.13 |
| Ph4-18 | 59 | 0 | 19 | 1181 | 40 | 0.00 | 0.98 | 0.00 | 0.00 | 0.95 | 0.00 | 0.00 |
| Ph4-19 | 251 | 30 | 211 | 989 | 10 | 0.75 | 0.82 | 0.12 | 3.71 | 0.82 | 0.91 | 0.21 |
| Ph4-20 | 420 | 15 | 380 | 820 | 25 | 0.38 | 0.68 | 0.04 | 1.11 | 0.67 | 0.55 | 0.07 |
| Ph4-21 | 41 | 0 | 1 | 1199 | 40 | 0.00 | 1.00 | 0.00 | 0.00 | 0.97 | 0.00 | 0.00 |
| Ph4-22 | 42 | 2 | 2 | 1198 | 38 | 0.05 | 1.00 | 0.05 | 1.48 | 0.97 | 0.05 | 0.09 |
| Ph4-23 | 595 | 23 | 555 | 645 | 17 | 0.58 | 0.54 | 0.04 | 1.20 | 0.54 | 1.07 | 0.07 |
| Ph4-24 | 40 | 0 | 0 | 1200 | 40 | 0.00 | 1.00 | 0.00 | 0.00 | 0.97 | 0.00 | 0.00 |
| Ph4-25 | 161 | 5 | 121 | 1079 | 35 | 0.13 | 0.90 | 0.03 | 0.96 | 0.87 | 0.14 | 0.06 |
| Ph4-26 | 220 | 15 | 180 | 1020 | 25 | 0.38 | 0.85 | 0.07 | 2.11 | 0.83 | 0.44 | 0.13 |
| Ph4-27 | 148 | 12 | 108 | 1092 | 28 | 0.30 | 0.91 | 0.08 | 2.51 | 0.89 | 0.33 | 0.15 |
| Ph4-28 | 680 | 19 | 640 | 560 | 21 | 0.48 | 0.47 | 0.03 | 0.87 | 0.47 | 1.02 | 0.05 |
| Ph4-29 | 439 | 14 | 399 | 801 | 26 | 0.35 | 0.67 | 0.03 | 0.99 | 0.66 | 0.52 | 0.06 |
| Ph4-30 | 86 | 6 | 46 | 1154 | 34 | 0.15 | 0.96 | 0.07 | 2.16 | 0.94 | 0.16 | 0.13 |
| Ph4-31 | 761 | 25 | 721 | 479 | 15 | 0.63 | 0.40 | 0.03 | 1.02 | 0.41 | 1.57 | 0.06 |
| Ph4-32 | 214 | 14 | 174 | 1026 | 26 | 0.35 | 0.86 | 0.07 | 2.03 | 0.84 | 0.41 | 0.12 |
| Ph4-33 | 319 | 13 | 279 | 921 | 27 | 0.33 | 0.77 | 0.04 | 1.26 | 0.75 | 0.42 | 0.08 |
| Ph4-34 | 148 | 10 | 108 | 1092 | 30 | 0.25 | 0.91 | 0.07 | 2.09 | 0.89 | 0.27 | 0.13 |
| Ph4-35 | 585 | 21 | 545 | 655 | 19 | 0.53 | 0.55 | 0.04 | 1.11 | 0.55 | 0.96 | 0.07 |
| Ph4-36 | 450 | 13 | 410 | 790 | 27 | 0.33 | 0.66 | 0.03 | 0.90 | 0.65 | 0.49 | 0.06 |
| Ph4-37 | 670 | 27 | 630 | 570 | 13 | 0.68 | 0.48 | 0.04 | 1.25 | 0.48 | 1.42 | 0.08 |
| Ph4-38 | 270 | 17 | 230 | 970 | 23 | 0.43 | 0.81 | 0.06 | 1.95 | 0.80 | 0.53 | 0.12 |
| Ph4-39 | 749 | 26 | 709 | 491 | 14 | 0.65 | 0.41 | 0.03 | 1.08 | 0.42 | 1.59 | 0.07 |
| Ph4-40 | 625 | 16 | 585 | 615 | 24 | 0.40 | 0.51 | 0.03 | 0.79 | 0.51 | 0.78 | 0.05 |
| Ph4-41 | 979 | 36 | 939 | 261 | 4 | 0.90 | 0.22 | 0.04 | 1.14 | 0.24 | 4.14 | 0.07 |
| Ph4-42 | 650 | 34 | 610 | 590 | 6 | 0.85 | 0.49 | 0.05 | 1.62 | 0.50 | 1.73 | 0.10 |
| Ph4-43 | 768 | 26 | 728 | 472 | 14 | 0.65 | 0.39 | 0.03 | 1.05 | 0.40 | 1.65 | 0.07 |
| Ph4-44 | 959 | 39 | 919 | 281 | 1 | 0.98 | 0.23 | 0.04 | 1.26 | 0.26 | 4.16 | 0.08 |
| Ph4-45 | 396 | 23 | 356 | 844 | 17 | 0.58 | 0.70 | 0.06 | 1.80 | 0.70 | 0.82 | 0.11 |
| Ph4-46 | 180 | 12 | 140 | 1060 | 28 | 0.30 | 0.88 | 0.07 | 2.07 | 0.86 | 0.34 | 0.13 |
| Ph4-47 | 163 | 15 | 123 | 1077 | 25 | 0.38 | 0.90 | 0.09 | 2.85 | 0.88 | 0.42 | 0.17 |
| Ph4-48 | 937 | 25 | 897 | 303 | 15 | 0.63 | 0.25 | 0.03 | 0.83 | 0.26 | 2.48 | 0.05 |
| Ph4-49 | 146 | 14 | 106 | 1094 | 26 | 0.35 | 0.91 | 0.10 | 2.97 | 0.89 | 0.38 | 0.18 |
| Ph4-50 | 755 | 33 | 715 | 485 | 7 | 0.83 | 0.40 | 0.04 | 1.35 | 0.42 | 2.04 | 0.08 |
| Ph4-51 | 971 | 32 | 931 | 269 | 8 | 0.80 | 0.22 | 0.03 | 1.02 | 0.24 | 3.57 | 0.06 |
| Ph4-52 | 792 | 32 | 752 | 448 | 8 | 0.80 | 0.37 | 0.04 | 1.25 | 0.39 | 2.14 | 0.08 |
| Ph4-53 | 582 | 26 | 542 | 658 | 14 | 0.65 | 0.55 | 0.04 | 1.38 | 0.55 | 1.19 | 0.09 |
| Ph4-54 | 756 | 24 | 716 | 484 | 16 | 0.60 | 0.40 | 0.03 | 0.98 | 0.41 | 1.49 | 0.06 |
| Ph4-55 | 656 | 19 | 616 | 584 | 21 | 0.48 | 0.49 | 0.03 | 0.90 | 0.49 | 0.98 | 0.06 |
| Ph4-56 | 519 | 11 | 479 | 721 | 29 | 0.28 | 0.60 | 0.02 | 0.66 | 0.59 | 0.46 | 0.04 |
| Ph4-57 | 738 | 25 | 698 | 502 | 15 | 0.63 | 0.42 | 0.03 | 1.05 | 0.43 | 1.49 | 0.07 |
| Ph4-58 | 262 | 8 | 222 | 978 | 32 | 0.20 | 0.82 | 0.03 | 0.95 | 0.80 | 0.25 | 0.06 |
| Ph4-59 | 932 | 31 | 892 | 308 | 9 | 0.78 | 0.26 | 0.03 | 1.03 | 0.27 | 3.02 | 0.06 |
| Ph4-60 | 627 | 20 | 587 | 613 | 20 | 0.50 | 0.51 | 0.03 | 0.99 | 0.51 | 0.98 | 0.06 |
| Ph4-61 | 45 | 1 | 5 | 1195 | 39 | 0.03 | 1.00 | 0.02 | 0.69 | 0.96 | 0.03 | 0.04 |
| Ph4-62 | 980 | 31 | 940 | 260 | 9 | 0.78 | 0.22 | 0.03 | 0.98 | 0.23 | 3.58 | 0.06 |
| Ph4-63 | 82 | 2 | 42 | 1158 | 38 | 0.05 | 0.97 | 0.02 | 0.76 | 0.94 | 0.05 | 0.05 |
| Ph4-64 | 348 | 12 | 308 | 892 | 28 | 0.30 | 0.74 | 0.03 | 1.07 | 0.73 | 0.40 | 0.07 |
| Ph4-65 | 348 | 12 | 308 | 892 | 28 | 0.30 | 0.74 | 0.03 | 1.07 | 0.73 | 0.40 | 0.07 |
| Ph4-66 | 315 | 10 | 275 | 925 | 30 | 0.25 | 0.77 | 0.03 | 0.98 | 0.75 | 0.32 | 0.06 |
| Ph4-67 | 885 | 29 | 845 | 355 | 11 | 0.73 | 0.30 | 0.03 | 1.02 | 0.31 | 2.45 | 0.06 |
| Ph4-68 | 505 | 18 | 465 | 735 | 22 | 0.45 | 0.61 | 0.04 | 1.10 | 0.61 | 0.73 | 0.07 |
| Ph4-69 | 753 | 26 | 713 | 487 | 14 | 0.65 | 0.41 | 0.03 | 1.07 | 0.41 | 1.60 | 0.07 |
| Ph4-70 | 404 | 11 | 364 | 836 | 29 | 0.28 | 0.70 | 0.03 | 0.84 | 0.68 | 0.39 | 0.05 |
| Ph4-71 | 61 | 5 | 21 | 1179 | 35 | 0.13 | 0.98 | 0.08 | 2.54 | 0.95 | 0.13 | 0.15 |
| Ph4-72 | 244 | 7 | 204 | 996 | 33 | 0.18 | 0.83 | 0.03 | 0.89 | 0.81 | 0.21 | 0.06 |
| Ph4-73 | 197 | 12 | 157 | 1043 | 28 | 0.30 | 0.87 | 0.06 | 1.89 | 0.85 | 0.35 | 0.11 |
| Ph4-74 | 446 | 30 | 406 | 794 | 10 | 0.75 | 0.66 | 0.07 | 2.09 | 0.66 | 1.13 | 0.13 |
| Ph4-75 | 222 | 23 | 182 | 1018 | 17 | 0.58 | 0.85 | 0.10 | 3.21 | 0.84 | 0.68 | 0.19 |
| Ph4-76 | 953 | 37 | 913 | 287 | 3 | 0.93 | 0.24 | 0.04 | 1.20 | 0.26 | 3.87 | 0.07 |
| Ph4-77 | 943 | 37 | 903 | 297 | 3 | 0.93 | 0.25 | 0.04 | 1.22 | 0.27 | 3.74 | 0.08 |
| Ph4-78 | 925 | 37 | 885 | 315 | 3 | 0.93 | 0.26 | 0.04 | 1.24 | 0.28 | 3.52 | 0.08 |
| Ph4-79 | 903 | 37 | 863 | 337 | 3 | 0.93 | 0.28 | 0.04 | 1.27 | 0.30 | 3.29 | 0.08 |
| Ph4-80 | 324 | 17 | 284 | 916 | 23 | 0.43 | 0.76 | 0.05 | 1.63 | 0.75 | 0.56 | 0.10 |
| Ph4-81 | 631 | 24 | 591 | 609 | 16 | 0.60 | 0.51 | 0.04 | 1.18 | 0.51 | 1.18 | 0.07 |
| Ph4-82 | 888 | 34 | 848 | 352 | 6 | 0.85 | 0.29 | 0.04 | 1.19 | 0.31 | 2.90 | 0.07 |
| Ph4-83 | 454 | 31 | 414 | 786 | 9 | 0.78 | 0.66 | 0.07 | 2.12 | 0.66 | 1.18 | 0.13 |
| Ph4-84 | 490 | 11 | 450 | 750 | 29 | 0.28 | 0.63 | 0.02 | 0.70 | 0.61 | 0.44 | 0.04 |
| Ph4-85 | 179 | 5 | 139 | 1061 | 35 | 0.13 | 0.88 | 0.03 | 0.87 | 0.86 | 0.14 | 0.05 |
| Ph4-86 | 66 | 3 | 26 | 1174 | 37 | 0.08 | 0.98 | 0.05 | 1.41 | 0.95 | 0.08 | 0.09 |
| Ph4-87 | 353 | 11 | 313 | 887 | 29 | 0.28 | 0.74 | 0.03 | 0.97 | 0.72 | 0.37 | 0.06 |
| Ph4-88 | 328 | 17 | 288 | 912 | 23 | 0.43 | 0.76 | 0.05 | 1.61 | 0.75 | 0.56 | 0.10 |
| Ph4-89 | 274 | 13 | 234 | 966 | 27 | 0.33 | 0.81 | 0.05 | 1.47 | 0.79 | 0.40 | 0.09 |
| Ph4-90 | 629 | 25 | 589 | 611 | 15 | 0.63 | 0.51 | 0.04 | 1.23 | 0.51 | 1.23 | 0.08 |
| Ph4-91 | 359 | 14 | 319 | 881 | 26 | 0.35 | 0.73 | 0.04 | 1.21 | 0.72 | 0.48 | 0.08 |
| Ph4-92 | 44 | 2 | 4 | 1196 | 38 | 0.05 | 1.00 | 0.05 | 1.41 | 0.97 | 0.05 | 0.09 |
| Ph4-93 | 82 | 3 | 42 | 1158 | 37 | 0.08 | 0.97 | 0.04 | 1.13 | 0.94 | 0.08 | 0.07 |
| Ph4-94 | 113 | 5 | 73 | 1127 | 35 | 0.13 | 0.94 | 0.04 | 1.37 | 0.91 | 0.13 | 0.08 |
| Ph4-95 | 1164 | 39 | 1124 | 76 | 1 | 0.98 | 0.06 | 0.03 | 1.04 | 0.09 | 15.39 | 0.06 |
| Ph4-96 | 1054 | 37 | 1014 | 186 | 3 | 0.93 | 0.16 | 0.04 | 1.09 | 0.18 | 5.97 | 0.07 |
| Ph4-97 | 724 | 36 | 684 | 516 | 4 | 0.90 | 0.43 | 0.05 | 1.54 | 0.45 | 2.09 | 0.09 |
| Ph4-98 | 426 | 28 | 386 | 814 | 12 | 0.70 | 0.68 | 0.07 | 2.04 | 0.68 | 1.03 | 0.12 |
| Ph4-99 | 140 | 38 | 100 | 1100 | 2 | 0.95 | 0.92 | 0.27 | 8.41 | 0.92 | 1.04 | 0.43 |
| Ph4-100 | 116 | 38 | 76 | 1124 | 2 | 0.95 | 0.94 | 0.33 | 10.16 | 0.94 | 1.01 | 0.49 |
| Ph4-101 | 123 | 39 | 83 | 1117 | 1 | 0.98 | 0.93 | 0.32 | 9.83 | 0.93 | 1.05 | 0.48 |
| Ph4-102 | 134 | 38 | 94 | 1106 | 2 | 0.95 | 0.92 | 0.28 | 8.79 | 0.92 | 1.03 | 0.44 |
| Ph4-103 | 113 | 38 | 73 | 1127 | 2 | 0.95 | 0.94 | 0.34 | 10.42 | 0.94 | 1.01 | 0.50 |
| Ph4-104 | 109 | 38 | 69 | 1131 | 2 | 0.95 | 0.94 | 0.35 | 10.81 | 0.94 | 1.01 | 0.52 |
| Ph4-105 | 264 | 39 | 224 | 976 | 1 | 0.98 | 0.81 | 0.15 | 4.58 | 0.82 | 1.20 | 0.26 |
| Ph4-106 | 225 | 38 | 185 | 1015 | 2 | 0.95 | 0.85 | 0.17 | 5.24 | 0.85 | 1.12 | 0.29 |
| Ph4-107 | 201 | 38 | 161 | 1039 | 2 | 0.95 | 0.87 | 0.19 | 5.86 | 0.87 | 1.10 | 0.32 |
| Ph4-108 | 162 | 38 | 122 | 1078 | 2 | 0.95 | 0.90 | 0.23 | 7.27 | 0.90 | 1.06 | 0.38 |
| Ph4-109 | **122** | **38** | **82** | **1118** | **2** | **0.95** | **0.93** | **0.31** | **9.66** | **0.93** | **1.02** | **0.48** |

**S4. Molecular docking simulation**

S4.1. Self-docking molecular docking validation

S4.1.1. Self-docking validation for VEGFR2


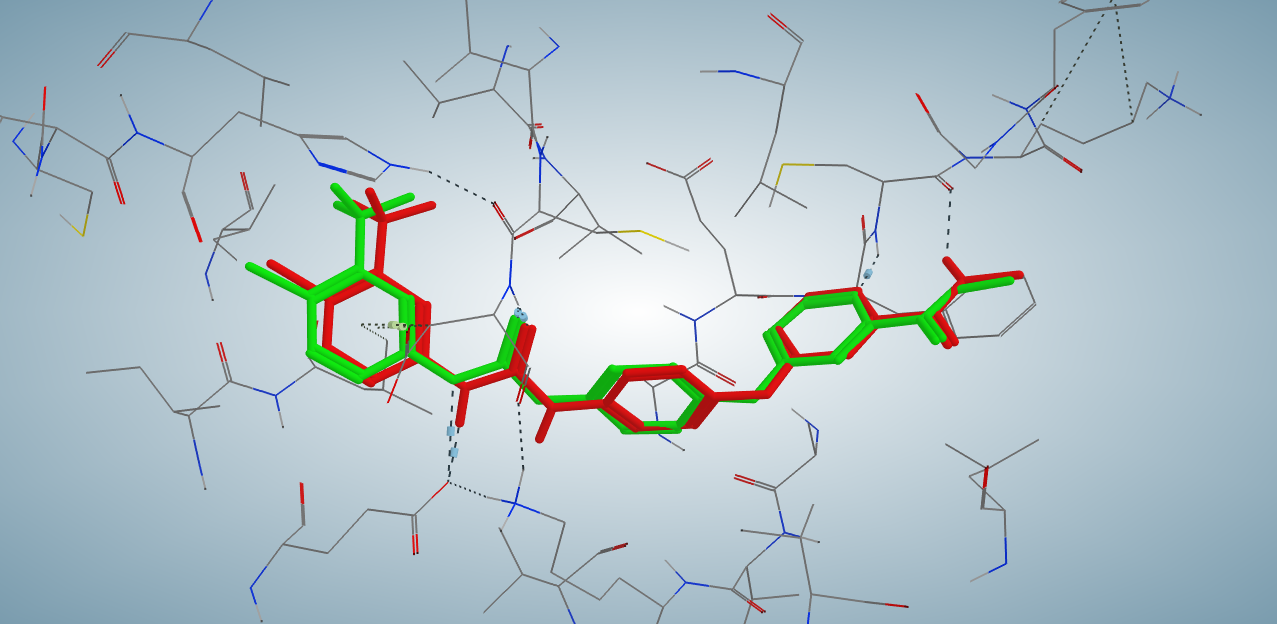


(A)


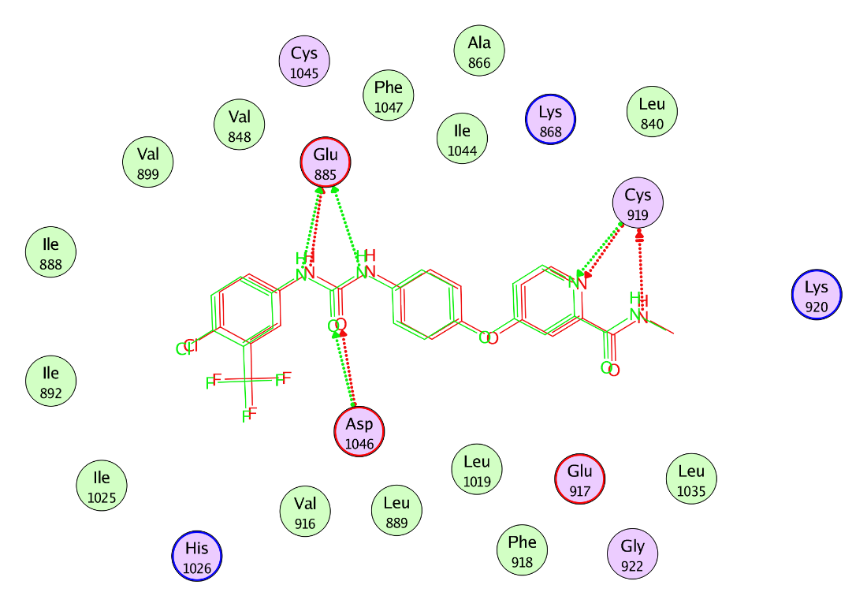


(B)


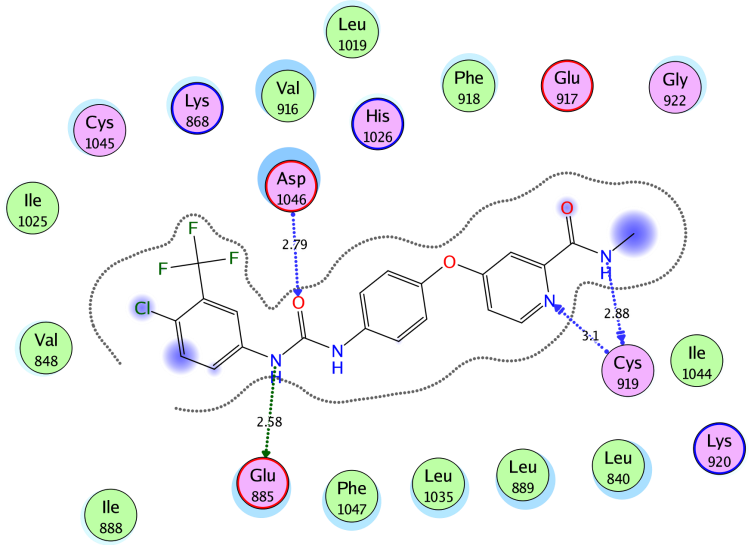


(C)

**Fig. S1.** 2D diagram (A) and 3D representation (B) of the superimposition of docking pose (green) and the co-crystallized (red) of sorafenib in the VEGFR-2 active site (C) 2D interaction diagram showing sorafenib docking pose interactions with the key amino acids (hot spots) in the VEGFR-2 active site. (Distances in Å).

S3.1.2. Self-docking validation for FAK


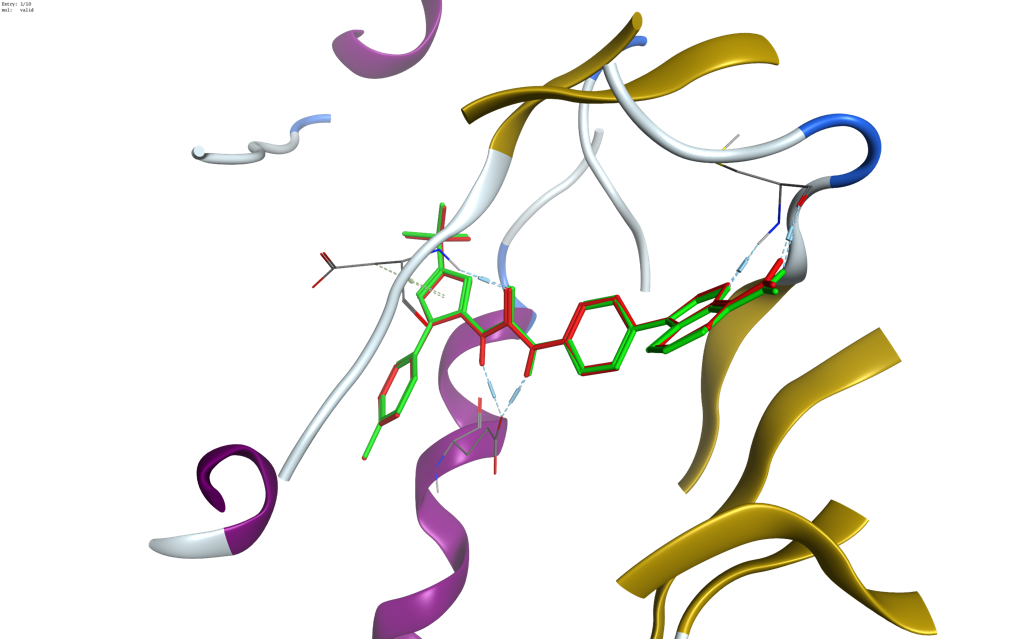


(A)


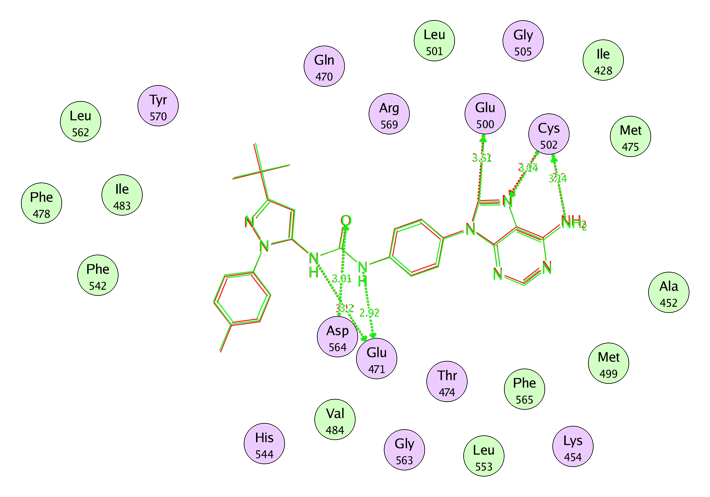


(B)


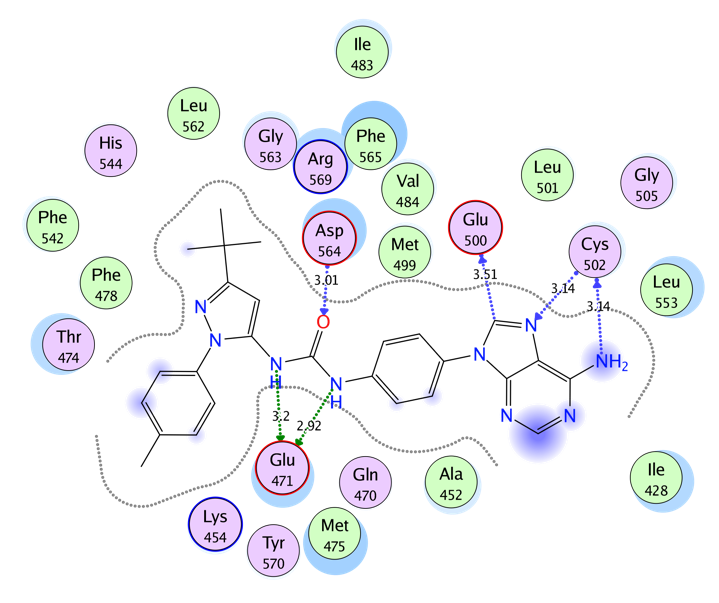


(C)

**Fig. S2.** 2D diagram (A) and 3D representation (B) of the superimposition of docking pose (green) and the co-crystallized (red) of co-crystalized FAK inhibitor in the FAK active site (C) 2D interaction diagram showing the FAK inhibitor docking pose interactions with the key amino acids (hot spots) in the FAK active site. (Distances in Å).

S4.2. Docking energy score (S) in kcal/mol for the common 13 compounds in VEGFR2 and FAK binding sites

Table S5. Docking energy score (S) in kcal/mol for the common 13 compounds in VEGFR2 and FAK binding sites

|  | VEGFR2 | FAK | Average |
| --- | --- | --- | --- |
| ZINC ID | Docking score  S (kcal/mol) | Docking score  S (kcal/mol) | Docking score  S (kcal/mol) |
| ZINC15444005 | −15.21 | −14.57 | −14.89 |
| ZINC55101214 | −15.65 | −11.55 | −13.60 |
| ZINC13324828 | −14.79 | −12.06 | −13.43 |
| ZINC91956669 | −13.16 | −13.41 | −13.29 |
| ZINC80154536 | −14.25 | −11.94 | −13.09 |
| ZINC92441642 | −13.49 | −12.34 | −12.91 |
| ZINC92388226 | −13.06 | −12.62 | −12.84 |
| ZINC78522361 | −12.86 | −12.72 | −12.79 |
| ZINC09874684 | −12.53 | −12.76 | −12.64 |
| ZINC92354322 | −12.79 | −12.47 | −12.63 |
| ZINC09875266 | −12.58 | −12.42 | −12.50 |
| ZINC23251457 | −12.89 | −11.95 | −12.42 |
| ZINC79747505 | −12.53 | −12.03 | −12.28 |
| Co-crystalized ligands | **−15.24** | **−16.02** | **−15.63** |

**References**

1. McTigue M, Murray BW, Chen JH, Deng Y-L, Solowiej J, Kania RS. Molecular conformations, interactions, and properties associated with drug efficiency and clinical performance among VEGFR TK inhibitors. Proceedings of the National Academy of Sciences. 2012;109:18281–9.

2. Potashman MH, Bready J, Coxon A, DeMelfi TM, DiPietro L, Doerr N, et al. Design, Synthesis, and Evaluation of Orally Active Benzimidazoles and Benzoxazoles as Vascular Endothelial Growth Factor-2 Receptor Tyrosine Kinase Inhibitors. J Med Chem. 2007;50:4351–73.

3. Oguro Y, Miyamoto N, Okada K, Takagi T, Iwata H, Awazu Y, et al. Design, synthesis, and evaluation of 5-methyl-4-phenoxy-5H-pyrrolo[3,2-d] pyrimidine derivatives: Novel VEGFR2 kinase inhibitors binding to inactive kinase conformation. Bioorg Med Chem. 2010;18:7260–73.

4. Cee VJ, Cheng AC, Romero K, Bellon S, Mohr C, Whittington DA, et al. Pyridyl-pyrimidine benzimidazole derivatives as potent, selective, and orally bioavailable inhibitors of Tie-2 kinase. Bioorg Med Chem Lett. 2009;19:424–7.

5. Okaniwa M, Hirose M, Imada T, Ohashi T, Hayashi Y, Miyazaki T, et al. Design and Synthesis of Novel DFG-Out RAF/Vascular Endothelial Growth Factor Receptor 2 (VEGFR2) Inhibitors. 1. Exploration of [5,6]-Fused Bicyclic Scaffolds. J Med Chem. 2012;55:3452–78.

6. Matsui J, Yamamoto Y, Funahashi Y, Tsuruoka A, Watanabe T, Wakabayashi T, et al. E7080, a novel inhibitor that targets multiple kinases, has potent antitumor activities against stem cell factor producing human small cell lung cancer H146, based on angiogenesis inhibition. Int J Cancer. 2008;122:664–71.

7. Okamoto K, Ikemori-Kawada M, Jestel A, von König K, Funahashi Y, Matsushima T, et al. Distinct Binding Mode of Multikinase Inhibitor Lenvatinib Revealed by Biochemical Characterization. ACS Med Chem Lett. 2015;6:89–94.

8. McAulay K, Hoyt EA, Thomas M, Schimpl M, Bodnarchuk MS, Lewis HJ, et al. Alkynyl Benzoxazines and Dihydroquinazolines as Cysteine Targeting Covalent Warheads and Their Application in Identification of Selective Irreversible Kinase Inhibitors. J Am Chem Soc. 2020;142:10358–72.

9. Grädler U, Bomke J, Musil D, Dresing V, Lehmann M, Hölzemann G, et al. Fragment-based discovery of focal adhesion kinase inhibitors. Bioorg Med Chem Lett. 2013;23:5401–9.

10. Gong C, Tan H, Zhang Q. Structure Optimization and Structure-Activity Relationship Study of a Kind of Type II FAK Inhibitors with N -Phenylpyrazole Ureas. Chinese Journal of Organic Chemistry. 2018;38:3086.

11. Bhattacharya SK, Aspnes GE, Bagley SW, Boehm M, Brosius AD, Buckbinder L, et al. Identification of novel series of pyrazole and indole-urea based DFG-out PYK2 inhibitors. Bioorg Med Chem Lett. 2012;22:7523–9.

12. Allen JG, Lee MR, Han C-YE, Scherrer J, Flynn S, Boucher C, et al. Identification of small molecule inhibitors of proline-rich tyrosine kinase 2 (Pyk2) with osteogenic activity in osteoblast cells. Bioorg Med Chem Lett. 2009;19:4924–8.

13. Iwata H, Imamura S, Hori A, Hixon MS, Kimura H, Miki H. Biochemical Characterization of TAK-593, a Novel VEGFR/PDGFR Inhibitor with a Two-Step Slow Binding Mechanism. Biochemistry. 2011;50:738–51.

14. Manley PW, Drueckes P, Fendrich G, Furet P, Liebetanz J, Martiny-Baron G, et al. Extended kinase profile and properties of the protein kinase inhibitor nilotinib. Biochimica et Biophysica Acta (BBA) - Proteins and Proteomics. 2010;1804:445–53.

15. El Kerdawy AM, Osman AA, Zaater MA. Receptor-based pharmacophore modeling, virtual screening, and molecular docking studies for the discovery of novel GSK-3β inhibitors. J Mol Model. 2019;25.
